# NiReject: toward automated bad channel detection in functional near-infrared spectroscopy

**DOI:** 10.1117/1.NPh.11.4.045008

**Published:** 2024-11-04

**Authors:** Christian Gerloff, Meryem A. Yücel, Lena Mehlem, Kerstin Konrad, Vanessa Reindl

**Affiliations:** a JARA Brain Institute II, Molecular Neuroscience and Neuroimaging (INM-11), Jülich Research Centre, Jülich, Germany; b University Hospital RWTH Aachen, Department of Child and Adolescent Psychiatry, Psychosomatics and Psychotherapy, Child Neuropsychology Section, Aachen, Germany; c University of Cambridge, Cambridge Centre for Data-Driven Discovery, Department of Applied Mathematics and Theoretical Physics, Cambridge, United Kingdom; d Boston University, Neurophotonics Center, Department of Biomedical Engineering, Boston, United States; e Massachusetts General Hospital, Harvard Medical School, MGH/HST Athinoula A. Martinos Center for Biomedical Imaging, Department of Radiology, Charlestown, Massachusetts, United States; f Nanyang Technological University, School of Social Sciences, Department of Psychology, Singapore

**Keywords:** functional near-infrared spectroscopy, bad channels, noisy channels, pruning, machine learning, signal quality

## Abstract

**Significance:**

The increasing sample sizes and channel densities in functional near-infrared spectroscopy (fNIRS) necessitate precise and scalable identification of signals that do not permit reliable analysis to exclude them. Despite the relevance of detecting these “bad channels,” little is known about the behavior of fNIRS detection methods, and the potential of unsupervised and semi-supervised machine learning remains unexplored.

**Aim:**

We developed three novel machine learning-based detectors, unsupervised, semi-supervised, and hybrid NiReject, and compared them with existing approaches.

**Approach:**

We conducted a systematic literature search and demonstrated the influence of bad channel detection. Based on 29,924 signals from two independently rated datasets and a simulated scenario space of diverse phenomena, we evaluated the NiReject models, six of the most established detection methods in fNIRS, and 11 prominent methods from other domains.

**Results:**

Although the results indicated that a lack of proper detection can strongly bias findings, detection methods were reported in only 32% of the included studies. Semi-supervised models, specifically semi-supervised NiReject, outperformed both established thresholding-based and unsupervised detectors. Hybrid NiReject, utilizing a human feedback loop, addressed the practical challenges of semi-supervised methods while maintaining precise detection and low rating effort.

**Conclusions:**

This work contributes toward more automated and reliable fNIRS signal quality control by comprehensively evaluating existing and introducing novel machine learning-based techniques and outlining practical considerations for bad channel detection.

## 1 Introduction

Functional near-infrared spectroscopy (fNIRS) is an emerging, noninvasive, optical neuroimaging technique that draws from the advantage of being applicable in a wide range of daily life settings, across different clinical and non-clinical populations, as well as different age groups. However, a primary challenge of using fNIRS is that the measured light intensity from source-detector pairs, termed channels, can be strongly affected by various noise sources, complicating the reliable analysis of neural activity.^1^^,^^2^

In fNIRS, noise typically originates from physiological, technical, or mechanical causes, including participant movement. A broad range of processing techniques tailored to attenuate noise has been developed.^3^^–^^7^ For example, filtering techniques aim to attenuate frequencies related to physiological noise and noise resulting from the optical measurement system.^4^ Short-separation regression aims to separate the cerebral hemodynamic response from confounding factors such as systemic physiological noise using specific source-detector pairs with short distances^8^^,^^9^ and multimodal approaches that utilize hardware such as acceleration sensors to correct for motion artifacts.^10^^,^^11^ Moreover, machine learning-based denoising has been proposed to learn representations that distinguish noise and the hemodynamic response within a signal.^12^^,^^13^ However, not all signals can be improved in their quality. Particularly technical and mechanical sources of noise can pose challenging phenomena in fNIRS signals. For instance, mechanical forces that cause a loss or reduction of contact between the optode and skull across a longer period of time can degrade an entire recording. In such cases, when signal quality is substantially diminished over long sequences or entire signals, reliable signal processing becomes very challenging or impossible. These channels that do not allow for reliable decomposition of brain and noise-related signal components are called “noisy channels” or “bad channels.”^14^ Although the effects of bad channels on subsequent analyses have, to the best of our knowledge, not been systematically explored yet, it is generally assumed that they can introduce a bias in the subsequent analysis, leading to potentially wrong scientific conclusions. Consequently, one of the first and crucial steps in fNIRS analysis is a quality control aimed at identifying and rejecting (or “pruning”) bad channels to avoid corruption of downstream analysis (see also Ref. 15).

Currently, one of the most prevailing approaches to identifying and excluding bad channels is based on manual expert assessment through an ex-post visual inspection of signal characteristics (“visual inspection,” e.g., Refs. 15 and 16). However, depending on the experience of expert raters, their given instructions, and their individual perception of signal quality, ratings may vary.^17^ Today, with an increasing number of optodes per device and growing sample sizes, this subjective approach has rendered it a costly and no longer practicable task. Hence, there is a pressing need in the field of fNIRS to develop practicable and precise methods for detecting bad channels in a more automated manner. To date, there has been very limited work on automatic bad channel detection for fNIRS, primarily focusing on thresholding-based approaches (reviewed in Sec. 1.1). Only a few studies have explored supervised machine learning-based detectors (reviewed in Sec. 1.2). In the following, we discuss their advantages and challenges and outline the potential of machine learning-based bad channel detection for fNIRS.

### 1.1 Established Approaches to Bad Channel Detection

The limitations of visual inspection have motivated attempts to base fNIRS bad channel detection on more automated, objective criteria.^18^ These methods are designed to detect bad channels using statistics particularly tailored to the properties of fNIRS signals (see Sec. 2.4.1). By manually specifying a fixed threshold for a single or multiple statistical metrics, a simple profile is created to differentiate between acceptable and aberrant data, such as the coefficient of variation (CoV).^19^ fNIRS typically measures light intensity changes at two wavelengths corresponding to changes in the concentration of oxyhemoglobin (HbO) and deoxyhemoglobin (HbR), thus some detection methods, such as the scalp coupling index (SCI)^20^, assess the coupling of these signals between wavelengths. Other metrics are based on the similarity of the signals across different brain regions or subjects,^21^ or they assess each signal independently of the others, e.g., Refs. 22–24. However, most metrics primarily capture a single or a few characteristics of the expected signal, so one metric may not be sufficient to accommodate the various causes of bad channels (see Sec. 3.3). A few studies combined distinct metrics, such as the CoV and signal power, to address this issue (e.g., Ref. 25). Similarly, the authors of Ref. 23 proposed the “placing headgear optodes efficiently before experimentation” algorithm (Phoebe),^23^ which combines the SCI and peak power. Other studies have extended this concept by combining metrics and expert ratings in a rule-based workflow.^26^^–^^28^ Similarly, Ref. 29 derived the signal quality index (SQI), a rule-based index based on thresholds calculated from previously rated data. However, all of these traditional approaches bear constraints. In addition to meaningful upper or lower boundaries, the specific choice of a fixed threshold remains arbitrary, depends on personal judgment, and may need to be adapted to experimental settings. A step forward was made by an fNIRS reporting tool,^30^ which visualizes the SCI and peak power^23^ across varying thresholds to increase researchers’ sensitivity to the results produced by the favored threshold. To optimize the threshold for the dataset at hand, the authors of Ref. 14 formulated an optimization problem that minimizes the loss between the threshold-based metric and the expert rating. However, a challenge that pertains to thresholding is often the high variance of detection performance and the high number of false positives, which limits its practicability.^31^^,^^32^

### 1.2 Potential of Machine Learning for Bad Channel Detection

Remarkable improvements in machine learning and increasing datasets have made these techniques an essential instrument for detecting aberrant data in medical imaging and beyond.^33^^–^^35^ These methods avoid manual thresholds and are more adaptive by capturing more complex patterns of the data than traditional rule-based approaches, leading to outstanding detection performances across various domains. To achieve this, machine learning-based detection methods aim to learn in an unsupervised (no manual rated data needed), semi-supervised (partly rated data needed), or supervised (high amount of rated data needed) fashion an effective representation separating acceptable and aberrant data.^36^^–^^38^ Thus, a key difference between machine learning-based detectors stems from the amount of “ground truth” data required, typically derived from manual expert ratings of the signals.

Unsupervised machine learning-based detectors such as isolation forest (IFOREST)^39^ are the predominant type of algorithms that have been developed in the machine learning community, and they have been tested for a variety of applications such as disease diagnosis, speech recognition, object recognition in imaging, or financial fraud detection but so far not in fNIRS bad channel detection.^35^^,^^36^ These detectors can be applied without any rating information, as is the case for thresholding-based approaches. Although unsupervised detection clearly benefits from low human effort, their performance is strongly determined by the extent to which their representation exploits the data characteristics that truly separate signals of acceptable quality and aberrant signals. This makes these detection methods fairly flexible in their application but may leave performance reserves that could be leveraged from expert raters’ experience.

Semi-supervised machine learning-based detectors utilize information from partially labeled datasets while maintaining the ability to detect unseen notions of signal anomalies, i.e., unrated bad channel variations.^36^ These detectors often enrich the partially labeled input data with representations or scores learned from unsupervised methods. For example, extreme gradient boosting outlier detection (XGBOD)^40^ ensembles the scores of unsupervised detectors, such as IFOREST, and feature encoding with autoencoders for weakly supervised anomaly detection (FEAWAD),^41^ builds on the latent representation from autoencoders. By this, semi-supervised detectors stem from the experience of expert raters to enhance the learned representations while being sensitive to various notions of bad channels that are not being rated. As for unsupervised ML-based detectors, the application of semi-supervised detectors remains an uncharted field in fNIRS.

Supervised machine learning-based detectors are trained on a training set to predict a class on an unseen test set, thus depending on a substantial proportion of labeled data. Importantly, this approach necessitates the presence of all notions of bad channels in the training data. During the past few years, two studies pioneered supervised machine learning approaches for fNIRS bad channel detection. Reference 42 performed the first bad channel detection using a machine learning-based classifier on an internally rated dataset (N=15). The authors trained a support vector machine (SVM)^43^ based on the SQI and compared its classification performance with that of SQI thresholding and a combined thresholding approach termed “placing headgear optodes efficiently before experimentation” (Phoebe),^23^ achieving superior performance. Reference 31 compared thresholding-based detection, using SCI, peak power (Sec. 2.4.1), and a version of the CoV, with an SVM, random forest, and a convolutional neural network using data of N=65 subjects. Both studies relied on a single, internally assessed dataset. Overall, the studies indicated potential performance benefits of machine learning-based detectors compared with established thresholding-based detection. However, because supervised methods require a large amount of rated data, they are rarely applied in other domains (see Refs. 34 and 35). For instance, the training of the supervised machine learning models depended on manually assessing 75% of all data.^31^ Such an extensive rating procedure imposes significant demands on human effort, making this approach expensive, strongly dependent on the decision of manual raters, and less scalable.

### 1.3 Challenges in the Adaptation and Development of fNIRS Bad Channel Detectors

Despite their potential for fNIRS, there are several challenges and limitations of machine-learning-based detectors to consider. Semi-supervised or unsupervised machine learning methods are arguably of greater practical relevance compared with supervised methods due to less required rating effort.^35^^,^^44^ However, most unsupervised, semi-supervised, and supervised machine learning approaches from other domains do not, or only to a limited degree, allow for the inclusion of prior knowledge, such as physically meaningful upper and lower boundaries, or do not consider that some detected channels may be of unexpectedly good rather than bad quality. Furthermore, practitioners require discrete and interpretable detection scores to make informed decisions, a demand unmet by current supervised detectors for fNIRS bad channel detection (see Refs. 17, 31, and 45). Although semi-supervised detectors appear attractive as a compromise between supervised and unsupervised methods, practical challenges of deciding how many and which signals to rate remain. Thus, a system that efficiently suggests a subset of signals for human feedback to subsequently perform semi-supervised detection, which is referred to as a “hybrid model,” is needed.

In addition to such application-driven challenges, the systematic comparison and methodological development of detection methods are challenged by the fact that the ground truth of bad channels is inherently unknown in real-world datasets. First attempts to assess a range of selected thresholding approaches and supervised detection for fNIRS bad channel detection are based solely on single in-house rated datasets serving as a ground truth, but a systematic evaluation of bad channel detectors across independently rated datasets is lacking (Sec. 1.2). Apart from expert ratings, synthetic generation mechanisms for bad channels may supplement real-world data with an objective assessment that enables more fine-grained insights into the detection performance under controlled conditions of various dataset characteristics, such as varying contamination and different bad channel phenomena.

### 1.4 Interpretable Machine Learning Detector and Framework for Systematic Bad Channel Detection in fNIRS

To fill these gaps, we developed NiReject, an interpretable machine learning method that detects bad channels *a posteriori* based on tail probabilities of multivariate cumulative distribution functions. The detector differs from existing approaches in its ability to account for prior information on meaningful feature distributions, provide interpretable and discrete detection scores, and be suitable for unsupervised and semi-supervised learning. We systematically assessed the performance of NiReject by comparing it with six of the most established thresholding-based detectors in fNIRS, five prominent distance- and density-based detectors, four unsupervised machine learning, and two semi-supervised machine learning detectors. We evaluated their performance first, using two independently rated, openly available fNIRS datasets and second, using simulated fNIRS signals with major bad channel phenomena. We evaluated the detectors’ robustness and stability under varying contamination rates, annotation errors of experts, and available ratings. Finally, we developed and evaluated a hybrid method for NiReject that includes an unsupervised step to identify specific channels for human feedback, followed by a semi-supervised training phase. To summarize, we aimed to investigate the performance, cost-efficiency, and practicability of different detection methods in the following questions:

Q1: How do established, unsupervised, and semi-supervised methods for bad channel detection perform across real-world datasets?
Q2: How do detection methods behave under different bad channel phenomena?
Q3: How do dataset characteristics, specifically contamination rate, rating effort, and rating errors, affect the detectors’ performances?
Q4: Can a hybrid detection method that integrates a human feedback loop overcome the practical challenges of semi-supervised detection?

## 2 Methods and Experiments

### 2.1 Formal Task Definition

Conceptually, bad channel detection aims to assign each channel a value indicating whether a particular channel should be considered for further analysis or rejected.

Thus, given a set of n spatio-temporal signals denoted by $X=\{X_{1},\ldots ,X_{n}\}$, the main objective of a bad channel detector D is to generate a detection score $S:=D(X)\in R^{n}$, where higher $s_{i}$ indicates that the signal i is more likely to be a bad channel than a lower score. Depending on the specific detection method, the detection score can either be continuous $s_{i}\in [0,1]$ or it can map X to a binary score with $s_{i}\in \{0,1\}$, i.e., as in threshold-based approaches.^46^ Let $Y\in R^{n}$ denote a binary vector of n labels, where $y_{i}\in \{0,1\}$ indicates either an acceptable ($y_{i}=0$) or a bad channel ($y_{i}=1$), and the detection performance of D is assessed by comparing the detection scores $S_{\text{test}}$ from a test set $X_{\text{test}}\in R^{m}$ against the corresponding label-signal pairs {X,Y}.

### 2.2 Datasets

#### 2.2.1 Real-world datasets

Detection performance was evaluated based on two publicly available fNIRS datasets (R22^26^ and N21^47^) that contain an expert rating. R22 consists of $N_{\text{signals}}=5984$ signals per wavelength ($\lambda_{1}=690\;\mathrm{nm}$, $\lambda_{2}=850\;\mathrm{nm}$) measured over k=22 prefrontal channels. Raw intensity measures were obtained from 34 children (M=14.26±2.206 years) and 68 adults (M=33.22±11.80 years) in two tasks with two runs per child and one run per adult, each with a sampling rate of 10 Hz using a continuous-wave fNIRS device (ETG-4000, Hitachi Medical Corporation, Japan). N21 consists of $N_{\text{signals}}=640$ signals per wavelength ($\lambda_{1}=760\;\mathrm{nm}$, $\lambda_{2}=850\;\mathrm{nm}$) measured over k=16 prefrontal and temporo-parietal channels. The dataset includes 20 children (M=5.4±0.125 years) and 20 adults (M=37.2±3.51 years), recorded with a sampling rate of 7.81 Hz using a different continuous-wave system (NIRSport, NIRx GmbH, Berlin, Germany). Records of both datasets include sequences of task-related data (subject performs a task) and resting data (subject is relaxing). R22 and N21 were independently rated by different research groups. N21 additionally used the average signal level during the rating procedure (see Sec. S1.1 in the Supplementary Material). The expert ratings Y code acceptable channels (y=0) and bad channels (y=1). The datasets vary in their ratio between acceptable and bad channels ($c_{R22}=4.22\%$, $c_{N21}=9.38\%$). The contamination per age group and dataset is depicted in Sec. S4.1 in the Supplementary Material. Notably, signals consisting only of missing values or only of zero amplitude were excluded from the datasets as their detection remains trivial.

#### 2.2.2 Synthetic datasets

We generated 14 synthetic datasets (S1 to S14 in the Supplementary Material). S1 to S13 focused on one of five bad channel phenomena each: atypical physiological oscillations across chromophores, temporal signal loss, and uni- and bidirectional shifts as well as spikes (see Sec. 4.3), with varying intensity rates. S1 to S13 consist of $N_{\text{signals}}=1600$ signals per wavelength with a sampling rate of 10 Hz from $N_{\text{subjects}}=100$ subjects using a common probe design provided by the Brain AnalyzIR Toolbox (16 frontal channels, $\lambda_{1}=690\;\mathrm{nm}$, $\lambda_{2}=830\;\mathrm{nm}$; see Ref. 48). In S14, we randomly composed each bad channel of multiple different bad channel phenomena instead of a single phenomenon and added nine additional short-distance channels ($N_{\text{signals}}=2500$). All 14 synthetic datasets consist of three rest and two task blocks with 20 stimuli each (as in real-world dataset R22, see Ref. 26). Further, each simulated dataset comprises 90% acceptable (y=0) and 10% bad channels (y=1), resulting in a contamination rate of $c_{\text{synthetic}}=10\%$. The data generation mechanism will be described in the following subsection. An overview of the characteristics of the two real-world and 14 synthetic datasets is provided in Table 1.

**Table 1:** Datasets.

Dataset	Type	$N_{\text{signals}}$	c	Characteristic	Experiment
R22	Real world	5984	4.22%	—	Q1, Q3
N21	Real world	640	9.38%	—	Q1
S1	Synthetic	1600	10%	Spikes, 6 n/min	Q2
S2	Synthetic	1600	10%	Spikes, 36 n/min	Q2
S3	Synthetic	1600	10%	Spikes, 60 n/min	Q2
S4	Synthetic	1600	10%	Unidirectional shifts, 12 n/min	Q2
S5	Synthetic	1600	10%	Unidirectional shifts, 24 n/min	Q2
S6	Synthetic	1600	10%	Unidirectional shifts, 36 n/min	Q2
S7	Synthetic	1600	10%	Bidirectional shifts, 12 n/min	Q2
S8	Synthetic	1600	10%	Bidirectional shifts, 24 n/n	Q2
S9	Synthetic	1600	10%	Bidirectional shifts, 36 n/min	Q2
S10	Synthetic	1600	10%	Signal loss, 10 n/min	Q2
S11	Synthetic	1600	10%	Signal loss, 50 n/min	Q2
S12	Synthetic	1600	10%	Signal loss, 100 n/min	Q2
S13	Synthetic	1600	10%	Atypical physiological	Q2
S14	Synthetic	2500	10%	Heterogeneous mixed, bad channel phenomena	Q4, preliminary

$N_{\text{signals}}$, number of signals in the dataset; c, contamination rate; n/min, number per minute.

### 2.3 Simulation of Acceptable fNIRS Signals and Bad Channel Phenomena

**Fig. 1:**
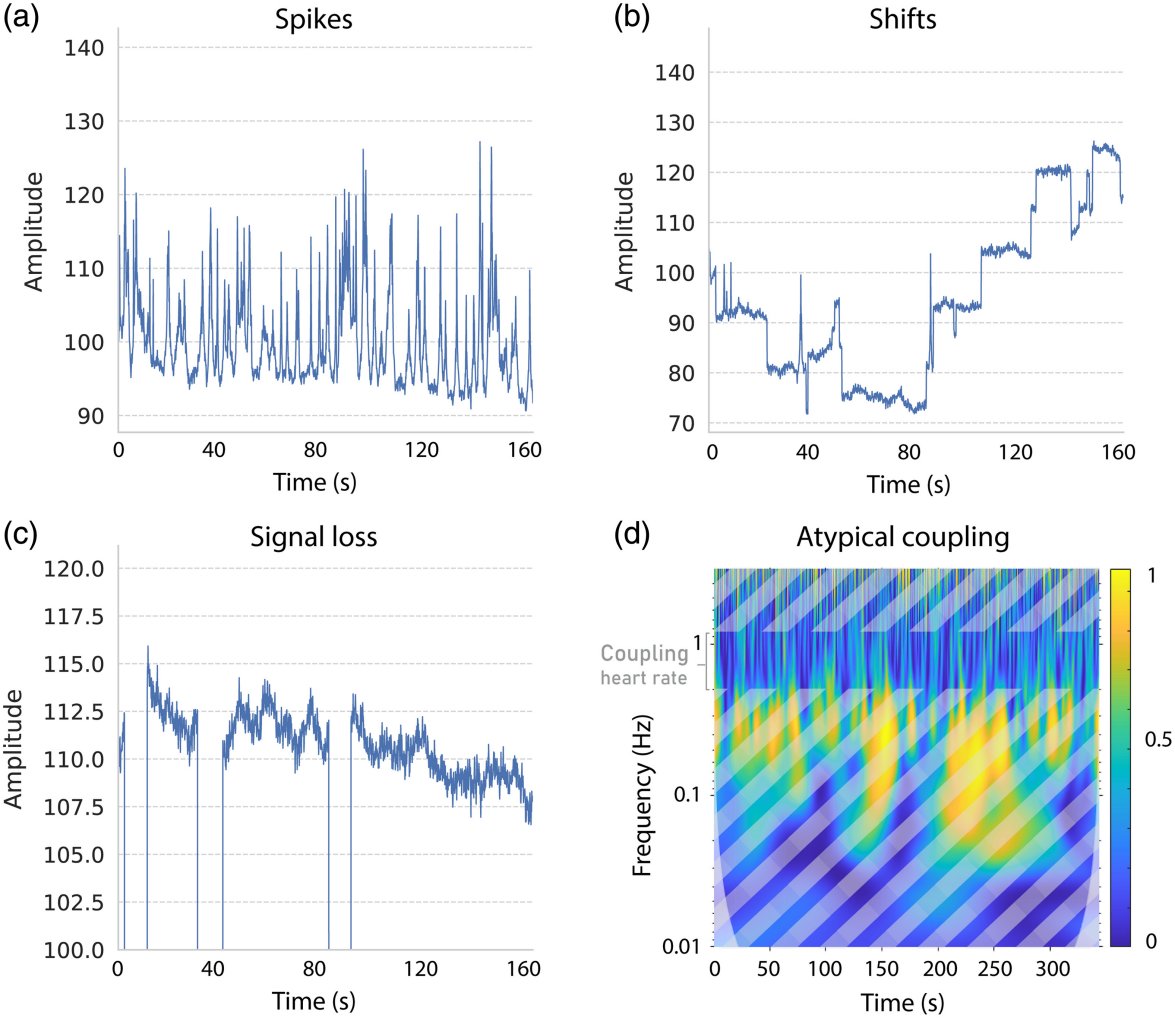
Bad channel phenomena in fNIRS: Four simulated bad channel phenomena are illustrated. (a) Spikes, (b) shifts, and (c) signal loss are represented using line plots. (d) The atypical coupling between wavelengths is visualized in the time-frequency space using wavelet coherence. Atypical coupling exhibits low coherence in cardiac-related frequencies.

To gain insights into the performance variability of detection methods, we simulated fNIRS signals composed of biophysiological oscillations, hemodynamic response, typical noise, and, in the case of bad channels, bad channel phenomena (see Fig. 1). These simulations are based on the functions of Refs. 48 and 49, with an adjusted generation mechanism described below.

#### 2.3.1 Signals of acceptable quality

For each subject, white noise was added as in the original implementation. The noise was spatially (σ=0.33)^48^ as well as temporally correlated using an autoregressive model (p=10), which falls between less (p=5)^50^ and more noisy settings (p=30).^1^ Further, physiological oscillations were added: heart-rate [f∈N∼(1.2  Hz,0.2  Hz)], respiration [f∈N∼(0.25  Hz,0.05  Hz)], and Mayer waves [f∈N∼(0.1  Hz,0.02  Hz)] with randomized phase [Δφ∈N∼(0.4,1)] (see also Refs. 49 and 51). Each signal was convolved with a canonical hemodynamic response function (as described in Ref. 48), encoding neural activation similar to the experimental design used in R22.^26^ Thus, the resulting signals of acceptable quality contain typical noise compositions that can be addressed by artifact correction techniques and do not require a rejection of the entire signal.

For a predefined proportion of signals ($c_{\text{synthetic}}=10\%$), the signal generation additionally modeled the following bad channel phenomena (s.t. y=1). All scenario parameters are provided in the artifact storage (see Sec. Code, Data, and Material).

#### 2.3.2 Atypical physiological oscillations across chromophores (dataset S13-S14)

Coupling mechanisms of the biophysiological systems, specifically the heart and brain, cause cardiac-related oscillations across signals of both wavelengths $\left(\Delta \varphi_{\text{physio}}=-0.5\right)$. Hence, an ideal detection method should be sensitive to signals with anomalous characteristics in cardiac-related frequencies. To test for this phenomenon, we varied the coupling between the physiological oscillation of the fNIRS signals of both wavelengths ($\Delta \varphi_{\text{physio}}=-0.7$).

#### 2.3.3 Temporal signal losses (dataset S10-S12, S14)

Various mechanical sources, such as vibrations or mechanical forces pulling the fNIRS cap, can result in a displacement of the optodes, causing a change or total loss of optical coupling. Longer signal losses cannot or can hardly be interpolated in a reliable manner. Here, we parametrized signal loss with 0 V amplitude by varying their duration (μ={1  s,5  s,10  s}, sd=0.2  s). Signal losses were jittered temporally across channels.

#### 2.3.4 Shifts (datasets S4-S9, S14)

In addition to signal loss, mechanical sources can also induce changes in optical coupling, leading to signal shifts. These shifts manifest as positive or negative amplitude changes. Although a few short signal shifts or single trends can usually be corrected using preprocessing techniques, a higher number of such shifts may distort the signal irreversibly. Shifts were simulated as instantaneous changes in the signal level that persist from the point of occurrence onward (see Ref. 49). Shifts were parametrized with an amplitude distribution N∼(4,2)^49^ and were varied in their occurrence (r={12  n/min,24  n/min,36  n/min}). Further, instantaneous changes were modeled either bi-directionally, which included both increased and decreased amplitudes within one signal, or unidirectionally, which included only increases or decreases in amplitude. All shifts were jittered temporally and in their amplitude across channels.

#### 2.3.5 Spikes (dataset S1-S13, S14)

Another phenomenon of mechanical causes is a sudden high-amplitude transient peak, also termed a spike. These mostly spatially nonuniform distributed anomalies can often be corrected during signal processing or in statistical downstream analysis.^1^ However, with an increasing number of spikes and varying durations, the assumptions for reliable downstream models may be violated, even after preprocessing. The amplitude of such spikes was parametrized with N∼(7,2)^49^ and duration N∼(0.2  s,0.1  s) given a sampling frequency of 10 Hz. Further, spikes were varied in their occurrence (r={6  n/min,36  n/min,60  n/min}) and jittered temporally and in their amplitude across channels.

### 2.4 Detection Methods

#### 2.4.1 Established detection methods in fNIRS

The most established detection methods in fNIRS typically employ a fixed threshold based on a metric derived from either raw light intensity or its derivatives, including raw attenuation/optical density, HbO, and HbR. The established thresholds that we used can be found in Table S4 in the Supplementary Material and in the artifact storage. A comprehensive overview, including other less frequently applied detectors, can be found in Sec. S1.1 in Supplementary Material.

##### Coefficient of variation (CoV)

The CoV^19^ is a widely applied, scale-invariant metric,^25^^,^^52^ which is calculated as the ratio between standard deviation and mean amplitude. Hence, an increasing CoV indicates a decreasing signal quality. A threshold is usually applied to the CoVs of both wavelengths and to their difference (e.g., Refs. 25 and 27). By this, the metric captures unexpected variability within the photo-detected signals and between both wavelengths. It is typically calculated based on the raw light intensity, independently for each channel and subject.^53^ In this study, the CoV was calculated independently for each channel based on raw intensity. *Utilized in toolbox*: LIONirs^53^ and nirsLAB. ^54^

##### Signal-to-noise-ratio (SNR)

The SNR^22^ has an inverse relationship with the CoV; thus, a lower SNR indicates decreasing signal quality (SNR∼1/CoV). It is often calculated by dividing the standard deviation of signals by their mean^22^ or median.^48^ Although the CoV and SNR might be used interchangeably, implementations based on the SNR often capture only the unexpected variability within photo-detected signals but not between signals of different wavelengths. Here, we used the mean of raw light intensity. *Utilized in toolbox*: Homer2&3^22^ and NIRS brain AnalyzIR toolbox.^48^

##### Scalp coupling index (SCI)

The SCI^20^ is defined as the zero-lag cross-correlation between both wavelengths of the optical density in cardiac-related frequencies, whereby higher values indicate better coupling. This is based on the assumption that synchronous cardiac pulsation of photo-detected signals is known to indicate good contact between optodes and scalp.^55^^,^^56^ The SCI can be derived either directly from the entire signals or from the median of windowed signals, which is less affected by the signal length. Therefore, we employed the latter implementation in this study. *Utilized in toolbox*: MNE-NIRS,^57^ NIRS brain AnalyzIR toolbox, Phoebe, and QT-NIRS.^30^

##### Peak power

Peak power,^23^ also termed the “peak spectral power,” judges bad channels based on the spectral power of the cross-correlated attenuation. Cardiac signals can be modeled with two sinusoidal waves, so the peak power mainly addresses spikes or baseline shifts that occur concurrently in both wavelengths in a predefined cardiac-related frequency spectrum. Again, we calculated the peak power based on the median of the windowed signals. *Utilized in toolbox*: MNE-NIRS, NIRS brain AnalyzIR toolbox, Phoebe, and QT-NIRS.

##### Placing headgear optodes efficiently before experimentation (Phoebe)

Placing headgear optodes efficiently before experimentation (Phoebe)^23^ is the name of a bad channel criterion composed of SCI and peak power. It detects a bad channel if either the SCI or peak power criterion is fulfilled. Thereby, it aims to prevent bias from unreasonably high SCI values, which can result from motion artifacts leading to synchronous peaks across wavelengths, utilized in the toolbox: Phoebe, NIRS brain AnalyzIR toolbox, and QT-NIRS.

Signal level, also termed the “average signal level” is a quality metric calculated from the mean amplitude of raw light intensity or from HbO/HbR concentration changes. This thresholding-based detection method rejects signals of low,^53^ high, or both low and high intensity.^22^ Thereby, it assumes that low intensity, e.g., from insufficient light penetration or scattering, and high light intensity, e.g., from ambient light sources, can affect fNIRS analysis. Here, we applied thresholds for both low and high concentration changes. *Utilized in toolbox*: Homer2&3, LIONirs,^53^ and NIRS brain AnalyzIR toolbox.

#### 2.4.2 Domain agnostic detection methods

Considering the progress in detection methods originating from other research areas, we incorporated the following prominent domain-agnostic detectors into the assessment of bad channel detection methods.

##### Distance- and density-based detectors (D&D)

D&D have a long-standing tradition as an effective detection technique. These detectors quantify the proximity of samples (here, channels) in an unsupervised manner based on the distance or density of neighboring samples.^38^^,^^58^ D&D are widely employed across research fields and are often considered precursors to machine learning-based detectors, thus rendering them a natural competitor. Specifically, we included the following prominent D&D: angle-based outlier detection^59^, k-nearest neighbors,^60^ local outlier factor,^61^ histogram-based outlier detection (HBOS),^62^ and minimum covariance determinant.^63^ For further methodological details and practical examples, see Refs. 38 and 58.

##### Unsupervised machine learning-based detectors

In the class of unsupervised machine learning-based detectors, we included the following representative algorithms: principal component analysis,^64^ cluster-based local outlier detection,^65^ one-class support vector machine,^66^ and IFOREST.^39^ Detailed descriptions of these algorithms can be found in Refs. 34, 38, and 67.

##### Semi-supervised machine learning-based detectors

Included prominent state-of-the-art detectors of this type are XGBOD^40^ and FEAWAD.^41^ More details can be found in Ref. 36.

### 2.5 Proposed Detectors: Unsupervised, Semi-Supervised, and Hybrid NiReject

#### 2.5.1 Unsupervised NiReject

**Fig. 2:**
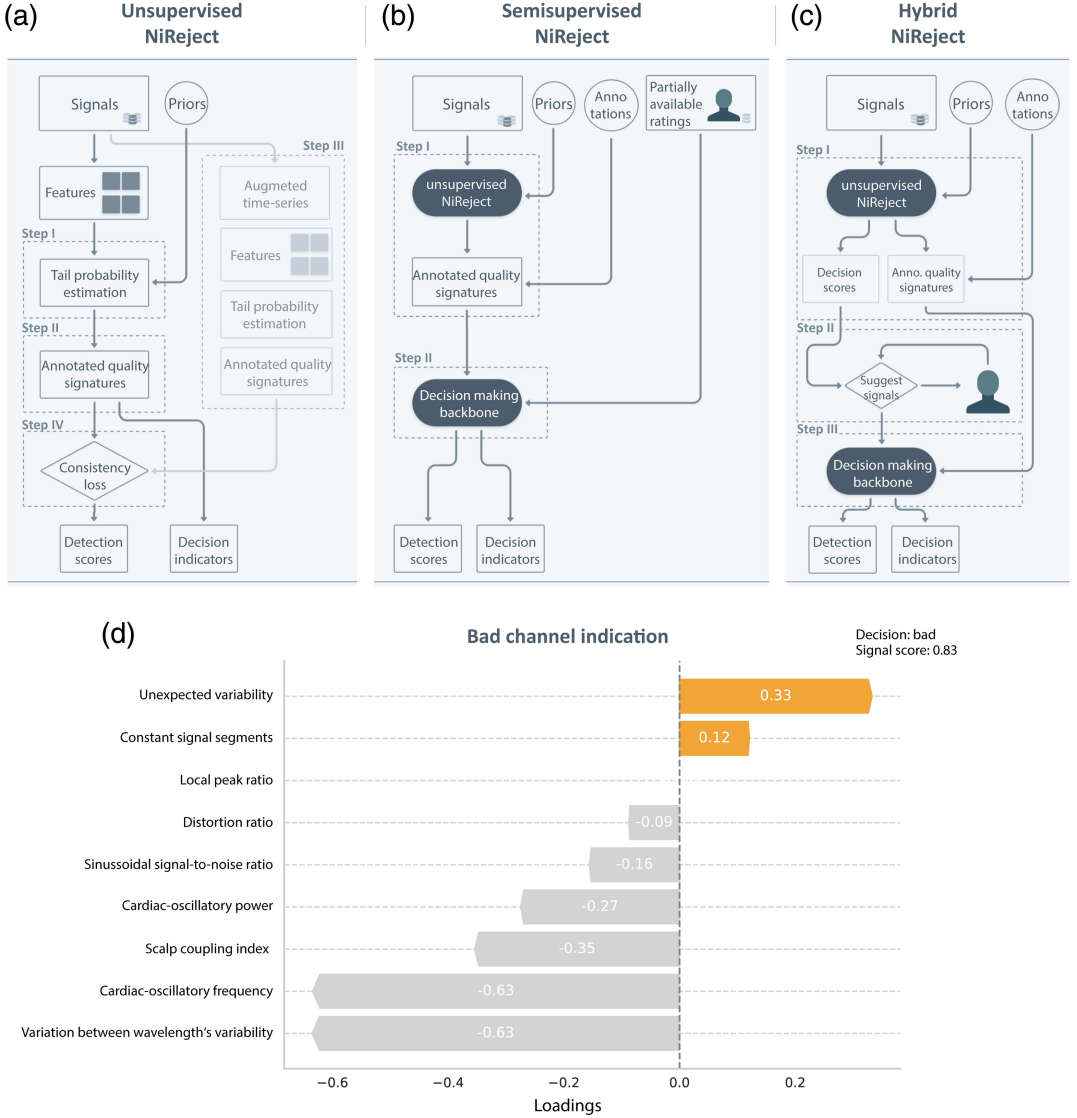
Illustration of the developed NiReject methods: For (a) unsupervised, (b) semi-supervised, and (c) hybrid NiReject, a schematic illustration of each algorithmic step is depicted. (d) The example of unsupervised NiReject’s quality indicators visualizes how NiReject provides insights into detection certainty and the characteristics on which the detector based its decision for a specific signal. The loading per characteristic can range between minus and plus one. Positive values indicate bad channel phenomena, and negative values close to minus one indicate a perfect behavior for a given characteristic.

Unsupervised NiReject follows a probabilistic approach by utilizing an empirical copula on given signal features to generate multivariate signatures of each neural signal without requiring any labels/ratings. Using these signatures, we can quantify the extent to which a signal deviates from the general distribution of signal characteristics. This methodology builds on previous work that stems from the idea that patterns characterizing aberrant data tend to appear in lower-density regions of a representation.^68^^,^^69^ The copula’s tail probability describes these lower-density regions. In other words, unsupervised NiReject assumes that patterns characterizing bad channels stand out because they occur less frequently. The detector uses informative priors to restrict the detection to relevant tails and subsequently discretize the detection probabilities by the alignment between augmented and original detection scores. In the following, we present step-by-step a more formal description of the detection method. Each step is illustrated schematically in Fig. 2(a).

Step I: *Estimation of tail probabilities*. Let the input feature space $Z\in R^{\text{nxd}}$ denote a set of d features and n samples derived from the original signals X. We define the priors $P\in R^{d}$ with p∈{−1,0,1} as input parameters of the detector D. According to Refs. 68 and 69, we denote the j’th entry of the vector $Z_{i}\in R^{n}$ as $Z_{i}^{\left(j\right)}$ and the univariate cumulative distribution function of a given feature j as $F^{\left(j\right)}:R\to [0,1]$. Following the approach of Refs. 68 and 69, the proposed detector estimates the tails of F as described in Ref. 68:
  F^left(j)(z)=1n∑i=1n1{Zi(j)≤z}, (1)
  F^right(j)(z)=1n∑i=1n1{Zi(d)>z}with  z∈R. (2)
  The resulting left- and right-tail probabilities, F^left(Zi) and F^right(Zi), can be derived from the factorization of F^left(d)(Zi(d)) and F^right(d)(Zi(d)) over the d’th dimension of Z, respectively, (see Sec. S2 in Supplementary Material). Often, it cannot be expected, or we have no prior knowledge, that a feature d tends to lie only in the left or right tail if it is associated with a bad channel. Therefore, in the work of Refs. 68 and 69, the coefficient of skewness is used to calculate the skewness-corrected tail probability $W(Z_{i})$.
Step II: *Calculation of quality signatures from confined tail probabilities*. The maximum across these three-tail probabilities ($W(Z_{i})$, F^left(Zi), and F^right(Zi)) served as the detection scores in previous work.^68^^,^^69^ However, not all extreme values in low-density regions may be meaningful. To prevent the erroneous detection of exceptionally good signals, NiReject extends previous approaches^68^^,^^69^ by confining the detection to a specific tail when prior knowledge is available. Specifically, the detector considers the informative priors $P\in R^{d}$ as input parameters, and p∈{−1,0,1} describes the selection of left (p=−1), right (p=1), or automatically estimated (p=0) probability. For p=0, the feature- and sample-specific scores are determined by the detector as
  V(j)(Zi(j))=max(−log(F^left(j)(Zi(j))),−log(F^right(j)(Zi(j))),W(j)(Zi(j))). (3)
  From this, the feature-specific quality signatures $H\in R^{nxd}$ can be generated and subsequently aggregated to obtain the signal-specific signatures $O\in R^{n}$ that characterize a bad channel as
  H(j)(Zi(j))=−1{pd<0}log(F^left(j)(Zi(j)))−1{pj≥0}log(F^right(j)(Zi(j)))+1{pj=0}V(j)(Zi(j)), (4)
  $$O(Z)=T(\overset{d}{\underset{j=1}{\sum }}H^{\left(j\right)}(Z_{i}^{\left(j\right)})).$$ (5)
  From Eq. (4), the detector provides interpretable insights for each signal i, delineating the extent to which the j’th feature contributes toward a bad channel [see Fig. 2(d)]. The transformation function T, as described in Ref. 70, is used to map H to a unified score ranging from zero to one. Thereby, O indicates whether i is more likely to be a bad channel.
Step III: *Augmented quality signatures*. The detector repeats step I to II on the augmented feature space $Z^{*}\in R^{nxd}$. This space is created by applying an augmentation function (A:R→R) to the original signals. The rationale is that an augmentation technique that approximately preserves key characteristics of the original data allows for covering an unexplored input space while leading to similar detection outcomes ($S(Z)=S(Z^{*})$). Because Z is specifically informed by the signal’s linear structure and amplitude distribution, the amplitude-adjusted Fourier transform^71^ is employed as an augmentation function in NiReject.
Step IV: *Deterministic detection scores*. To provide a deterministic output, the percentile Q *with* α∈(0,1) is used by the binary score function S, defined as
  $$S(O(Z_{i}),\alpha )=1\{O(Z_{i})\geq Q(O(Z),100(1-\alpha )))\}.$$ (6)

Because the actual contamination rate c∈[0,1] and consequently α are unknown in practice, the detector optionally employs the augmented signatures to determine α. Therefore, the consistency loss L based on the weighted cross entropy between both models is employed as

$$L=\overset{n}{\underset{i=1}{\sum }}\frac{1}{\alpha }S(O\left(Z\right),\alpha )O(Z^{*})-(\frac{1-S(O(Z),\alpha )}{1-\alpha })*(1-O(Z^{*})).$$ (7)

By learning α^ that minimizes L, the detector derives the consistency-based detection score S(H¯(Z),α^) with $S_{i}\in \{0,1\}$.

#### 2.5.2 Semi-supervised NiReject

Semi-supervised NiReject builds upon unsupervised NiReject [see Fig. 2(b)]. This detector is trained on the entire set of signals X and a subset of labels $Y^{l}\subseteq Y$. The partially available labels $Y^{l}$ encode only some bad channels of the total dataset, as described by $\gamma =\sum Y^{l}/\sum Y$, and some acceptable channels.

Step I: *Unsupervised representation learning*. In the first phase, the quality signatures H are generated by applying steps I and II from unsupervised NiReject on the entire feature space Z. Further, the detector enables annotating these with the spatial information (channel index) $C\in N^{k}$, leading to a representation $[C,H]\in R^{nx(d+1)}$. By this, the detector produces an unsupervised representation of the original signals that aims to encode signatures indicative of bad channels with respect to their location.
Step II: *Detection of bad channels*. Once the representation is learned, a semi-supervised detector D trains a decision model on this unsupervised representation along with the partially available labels $Y^{l}$, $\left\{[C,H],Y^{l}\right\}$. Similar to XGBOD,^40^ XGBoost^72^ is used as the default decision-making backbone of NiReject. This is motivated by the highly imbalanced class distribution that is typical for detection tasks and makes most classification methods impractical. Tree boosting algorithms, specifically XGBoost, provide a scalable approach that is well suited for high dimensional tabular data and imbalanced settings.^36^^,^^40^ The trained detector outputs S=D(Z).

#### 2.5.3 Hybrid NiReject

The hybrid machine learning system integrates an expert feedback loop into the detection process, as illustrated in Fig. 2(c).

Step I: *Unsupervised detection*. Initially, the implementation of unsupervised NiReject computes O and the binary detection scores $S_{\text{step}\;I}$ on the entire feature space Z.
Step II: *Human feedback loop*. In step II, the detector suggests a signal i to a human expert to retrieve rating information $\left\{X_{i},y_{i}\right\}$. Therefore, signals are sorted by high, medium, and lower O and then presented in an alternating order. To prevent a rater from recognizing this pattern, the sequence of these signals is randomly permuted within each batch of 12 signals. The detector suggests signals until the ratio of signals rated as bad and initially detected as bad reaches the predefined value t∈R, so that $\gamma_{h}\geq t$ with $\gamma_{h}=\overset{h}{\underset{i}{\sum }}y_{i}/\overset{n}{\underset{i}{\sum }}S_{\text{step}\;I,i}$. Through this mechanism, the detector efficiently acquires a rating $Y^{l}$ of a fraction of signals to train in step II a decision-making classifier. It should be noted that the choice of t can be guided by γ obtained in the AUC-PR findings in Sec. 3.4.2, e.g., t≙30%.
Step III: *Semi-supervised detection*. In step III, semi-supervised NiReject is trained on Z alongside the human ratings $Y^{l}$ final detection score.

### 2.6 Empirical Evaluation

#### 2.6.1 Parameter setting and features

For all detection methods, their default parameters were used and reported in the artifact storage accompanying this work. Although thresholding-based detectors directly operate on input signal X, NiReject and domain-agnostic detectors consider a given input feature space Z. To ensure a fair comparison, we considered the following well-established signal metrics as input features (Z) for NiReject and all domain-agnostic detectors.

##### Sinusoidal signal-to-noise and distortion ratio

Using a Kaiser window, we estimated the sinusoidal signal-to-noise ratio and harmonic distortion based on the assumption that the signal carries sinusoidal oscillations. These features are generic quality measures applied across domains, e.g., Refs. 73 and 74.

##### Local peak ratio

To capture the signal’s variance within raw attenuation, we considered the relative number of local peaks. Quadratic interpolation was applied to ensure a complete and smoother signal with reduced high-frequency noise. Subsequently, local maxima of the signal’s amplitude were identified. If a sample’s amplitude exceeded that of its neighboring samples, it was considered a local peak. We used the relative instead of absolute number of local peaks as a generic measure independent of the signal length.

##### CoV

The CoV and its difference between both wavelengths were included (Sec. 2.4.1).

##### Scalp coupling index

Scalp coupling was incorporated via the autocorrelation of the windowed optical densities (Sec. 2.4.1).

##### Cardiac-oscillatory modeled peak power and center frequency

In addition to established metrics, we incorporated the power spectrum of the periodic, cardiac-oscillatory components in fNIRS signals accounting for the individual cardiac-related frequency. The traditional peak power metric is based on a narrowband analysis and thereby requires precise predefined frequency bands for cardiac-related frequencies. However, heart-rate varies by task and subject’s age, making the specification of a narrow frequency band challenging. To overcome these limitations, we employed FOOOF.^75^ By this, we extracted the nearest peak power of the oscillatory component as well as its corresponding center frequency without the need to predefine a narrow frequency band.

#### 2.6.2 Test sets

In line with previous work on detection benchmarks (e.g., Refs. 36, 68, 69, and 76), we employed a stratified train-test split procedure to evaluate each detector on the test sets. Specifically, the sampling procedure reserves 60% of each dataset for training and allocates 40% for testing while preserving the proportion of bad channels to acceptable signals between the train and test data. This stratification mitigates potential biases resulting from distribution drifts between the train and test sets. We repeated each evaluation procedure 10 times with varying seeds and shuffled inputs to account for further sources of potential variance. The train-test set procedure is commonly applied in benchmarking detection methods because it allows for consistent assessment across learning regimes. It should be noted that we had to adjust this procedure to include a human feedback loop in experiment Q4 (Sec. 2.6.7). For real-world datasets, the manual rating decisions {0,1} of expert raters served as the “ground truth.” In simulated datasets, the ground truth is *a priori* known.

#### 2.6.3 Performance metrics

As each performance metric emphasizes different aspects of a model, we focused on metrics that are aligned with the experiment-specific research question in the main text.^77^ As a high number of false positives would demand manual reassessment, it is of high practical relevance to assess true bad channels over perceived bad channels. Hence, precision served as the main performance metric, emphasizing the importance of “being right” when identifying bad channels while penalizing for false positive detections. Precision is well suited for discrete detection scores and highly imbalanced cases as is often the case in bad channel detection. In addition, we reported ROC-AUC, another widely used metric for similar tasks, which balances the cost of false positives and false negatives more equitably. However, this measure should be interpreted with caution as it can be biased in highly imbalanced settings and is less sensitive to variation in the number of true bad channels.^67^ An ROC-AUC above the chance level indicates the general feasibility of a detection method to separate channels.

Precision and ROC-AUC, two widely applied and complementary performance metrics (see Ref. 67), served as our main performance measures in all analyses except when varying the rating information to assess the performance boundaries of the detectors in experiments of Q3 in Sec. 2.6.6. Here, we assessed whether the precision-recall trade-off, measured by precision-recall AUC (AUC-PR), reached an asymptotic behavior at a particular critical value; results for ROC-AUC and precision can be found in the Supplementary Material. Such performance boundaries may not be properly covered by precision or ROC-AUC, e.g., as it can be expected that precision increases monotonically when more evaluation information is available, but it is not said that, at the same time, the proportion of retrieved bad channels increases (Fig. S10 in the Supplementary Material). The AUC-PR was implemented by calculating the weighted mean between precision and recall, also termed the average precision score. This formulation is more conservative than AUC-PR using the trapezoidal rule as it avoids linear interpolation that can be too overoptimistic. For all other experiments, the AUC-PR is reported in the Supplementary Material.

#### 2.6.4 Experiment Q1: performance on real-world data

Detection performances on the real-world datasets, R22 and N21, were systematically evaluated based on the expert ratings made available by the public datasets.

For all performance metrics, the mean and standard deviations across 10 trials (sampling repetitions) were reported in the main tables [Fig. 4(a); all detailed performance metrics are available in the artifact storage]. Following benchmarking studies that compare methods across datasets (e.g., Refs. 36 and 78), results were additionally illustrated in critical difference diagrams^79^ [Figs. 4(b) and 4(c)]. For this, we utilized a Bayesian signed-rank test that enables the consideration of only performance differences of practical relevance as small differences may be evident but not necessarily meaningful.^80^ Specifically, a difference in performance metrics between two detectors was considered evident if one detector’s metric surpasses that of another by more than 0.01 (e.g., in precision) with a probability of 90% (credibility interval: CI = 90%, region of practical equivalence: ROPE=[−0.01,0.01]).

#### 2.6.5 Experiment Q2: variations of bad channel phenomena

We assessed how detection methods behave under different bad channel phenomena using synthetic datasets S1 to 13, each focusing on a single bad channel phenomenon with varying intensity (see Table 1). It should be noted that this experiment is less suited for semi-supervised detectors as they would be trained and tested on a single nuanced bad channel phenomenon, which is a relatively simple task for this type of learning regime (see results in Sec. S5 in Supplementary Material).

#### 2.6.6 Experiment Q3: variations of dataset characteristics

##### Varying contamination rates

To assess the effects of varying contamination rates on the detectors’ performances, we followed the procedure established in Refs. 36, 81, and 82 and varied the number of bad channels by up- and down-sampling them in a real-world dataset. We used R22 for all experiments of Q3 as it is larger in size than N21. This allowed us to estimate performance profiles across a broad range of contamination rates (c={1,…,50}) on real-world data including heterogeneous bad channels. This experiment focuses on NiReject and domain-agnostic detectors as their performances can vary with the contamination rate, whereas the decision function of thresholding-based detectors is independent of the contamination rate.

##### Varying available rating information

Detection methods that incorporate rating information during training, such as semi-supervised detectors, may be sensitive to the ratio of rated bad channels available during training to all bad channels in the dataset (γ).^36^ Following the approach of Ref. 36, we varied γ within the range {∼0,…,60} in the training set of the larger real-world dataset R22. Thus, at γ=60%, the training set would include the maximum number of rated bad channels possible because the stratified sampling procedure (Sec. 2.6.2) reserves 60% of the data for training. By examining the AUC-PR curve across different γ, this experiment determined the boundaries beyond which the amount of rating information no longer substantially improves the detection performance of semi-supervised detectors. These boundaries indicate the rating effort required to exploit the detection capacities of a given semi-supervised detector and can be used to determine the threshold t of the hybrid detection system described in Sec. 2.5.3.

##### Robustness against annotation errors

Because expert judgments are inherently subjective and raters can make false decisions, this experiment investigated the impact of such annotation errors on semi-supervised detectors. To this end, we shuffled ratings of acceptable and bad channels in the larger real-world dataset R22 while maintaining the same contamination rate (see Refs. 36, 81, and 82). Specifically, we assessed annotation errors within the range {∼0,…,60} in the training set. The AUC-PR curve was used to determine the extent to which annotation errors have no substantial impact on semi-supervised detectors.

#### 2.6.7 Experiment Q4: assessment of the NiReject hybrid-detection system

Because a hybrid machine learning system is only relevant if it increases the performance of its underlying unsupervised detector, we compared the detection performance of hybrid NiReject against unsupervised NiReject, mainly using ROC-AUC and precision. Therefore, the experiment employed the simulated dataset S14, which comprises various bad channel phenomena occurring either individually or together within a signal. The evaluation procedure of Sec. 2.6.2 was adjusted because the hybrid workflow requires a feedback loop within the evaluation procedure. Based on the empirical results in Sec. 3.4.2, the human feedback loop of hybrid NiReject (step II) was exercised until t=30% was reached. To account for performance variability between raters, we assessed hybrid NiReject twice, each time using the feedback of a different expert rater.

## 3 Results

### 3.1 Preliminary: Current Practices and Impact of Bad Channel Detection on Subsequent Statistical Analyses

To illustrate the pivotal role of bad channel detection, we first demonstrated its impact on both individual and group-level effects using two widely applied statistical analysis methods: average waveform analysis and general linear model (GLM). Furthermore, we provided a comprehensive overview of the currently used detection approaches in fNIRS, which informed the selection of detection methods used for the subsequent evaluation.

#### 3.1.1 Impact

**Fig. 3:**
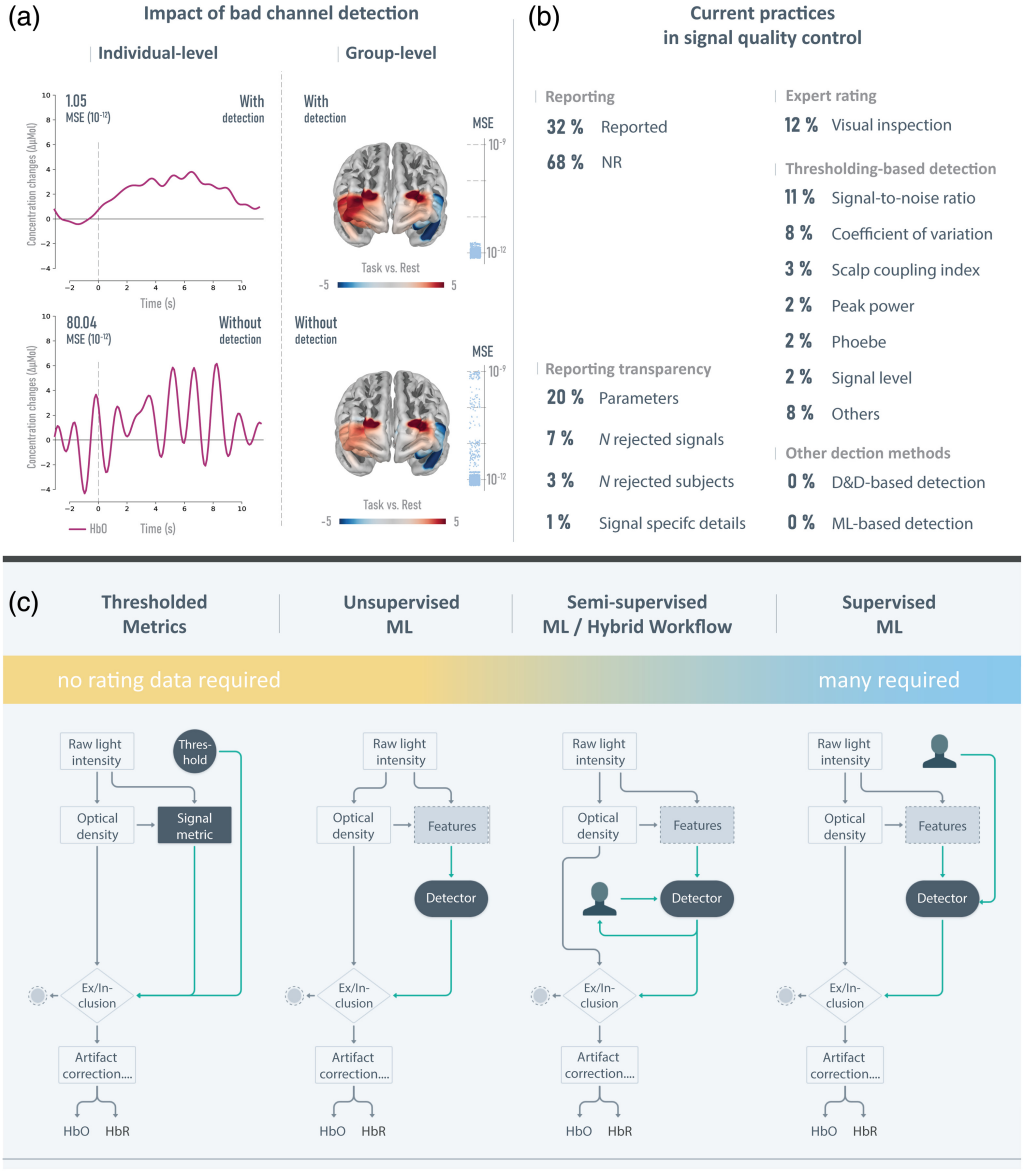
Bad channels detection in fNIRS: (a) Illustrates the effect of bad channel detection on average waveform analysis with a single subject (left, line chart) and group effects in GLM analysis of HbO (right, cortical map) using simulated fNIRS signals of 100 subjects. In all analysis regimes, state-of-the-art preprocessing techniques were applied. MSE between the fitted and simulated hemodynamic response is additionally displayed to quantify the impact of bad channel detection on subsequent analysis. Higher MSE implies lower alignment between ideal and estimated hemodynamic response function. Results with bad channel detection (top) differ from those without bad channel detection (bottom), as indicated by the MSE. (b) Shows the summary of the structured literature search on employed detection methods in fNIRS signal quality control. (c) Depicts the workflow for each class of detection methods and its dependence on required manual expert ratings, increasing from left to right. Each type of bad channel detection results in an exclusion or inclusion decision of an entire signal before subsequent processing techniques, such as artifact correction, can be applied.

The simulated dataset S14, comprised of an inhomogeneous mixture of bad channel phenomena, was utilized to illustrate the impact of bad channel detection. Both the average waveform analysis and the GLM analysis were performed on the entire dataset and on a subset of S14 containing no bad channels, which corresponds to an analysis with and without bad channel detection. The average waveform analysis of a single subject, as depicted in Fig. 3(a), revealed that the epoched task data follow the typical shape of a hemodynamic response function of HbO when a perfect bad channel detection method is applied. By contrast, the average waveform of a subject without effective bad channel detection is noticeably disturbed. An increased mean-square error (MSE) between the estimated and ideal hemodynamic response function, obtained from the subject’s signals without bad channel detection ($80.04*10^{-12}$) compared with the subjects’ signals after bad channel detection ($1.05*10^{-12}$) confirmed this result. However, one might assume that the impact of bad channels could be mitigated to some extent at the group level. Therefore, we employed a GLM to fit an ideal response function to the epoched data for each of the 100 subjects (see Ref. 83). The contrast between task and rest data on the group level was assessed using a linear mixed model for HbO. Results showed significant left lateral effects in frontal cortical regions when a precise bad channel detection was conducted, but these were not significant in the same analysis without bad channel detection. Specifically, among 16 long-distance channels, four no longer showed significance, two exhibited reduced significance, and one showed an increased p-value when compared with analysis in which no bad channel detection was applied (see Table S1 in the Supplementary Material). Importantly, the MSE across signals with and without bad channel detection varied by magnitudes. Further methodological details, including all statistical results, can be found in Sec. S2 in the Supplementary Material.

#### 3.1.2 Current practices

To provide a comprehensive overview of the current state of signal quality control in fNIRS, we conducted a structured literature search using the PRISMA-conform search engine SetYouFree.^84^ Consequently, we focused on the 100 most cited fNIRS studies published in 2022. The search results, depicted in Fig. 3(b), revealed that 68% of the studies did not report any bad channel detection. The most applied detection methods were visual inspection (12%), SNR (11%), and the CoV (8%). No machine learning methods and no unsupervised and semi-supervised machine learning were applied in the 100 most cited studies. An additional ex-post search found two recent studies that applied supervised machine learning detectors.^85^^,^^86^ The detection method’s parameters were reported in 20% of all publications. Only 7% reported the number of excluded signals, 3% reported the number of excluded subjects, and one study provided detailed information on the signals excluded (e.g., with respect to channel locations). The most often used toolbox for bad channel detection was Homer2 [Fig. S1(c) in the Supplementary Material], which utilizes the SNR and signal level (Sec. 2.4.1). Details of the search methodology and detailed search results can be found in Sec. S1 in the Supplementary Material. An overview of all detection methods is available in Tables S2 and S3 in the Supplementary Material.

### 3.2 Q1: Detection Performance on Real-World Datasets

Based on this literature search, we systematically assessed the performance of unsupervised and semi-supervised NiReject compared with the most established thresholding-based detection in fNIRS, distance- and density-based detectors as well as unsupervised and semi-supervised machine learning detectors using the open datasets N21 and R22

**Fig. 4:**
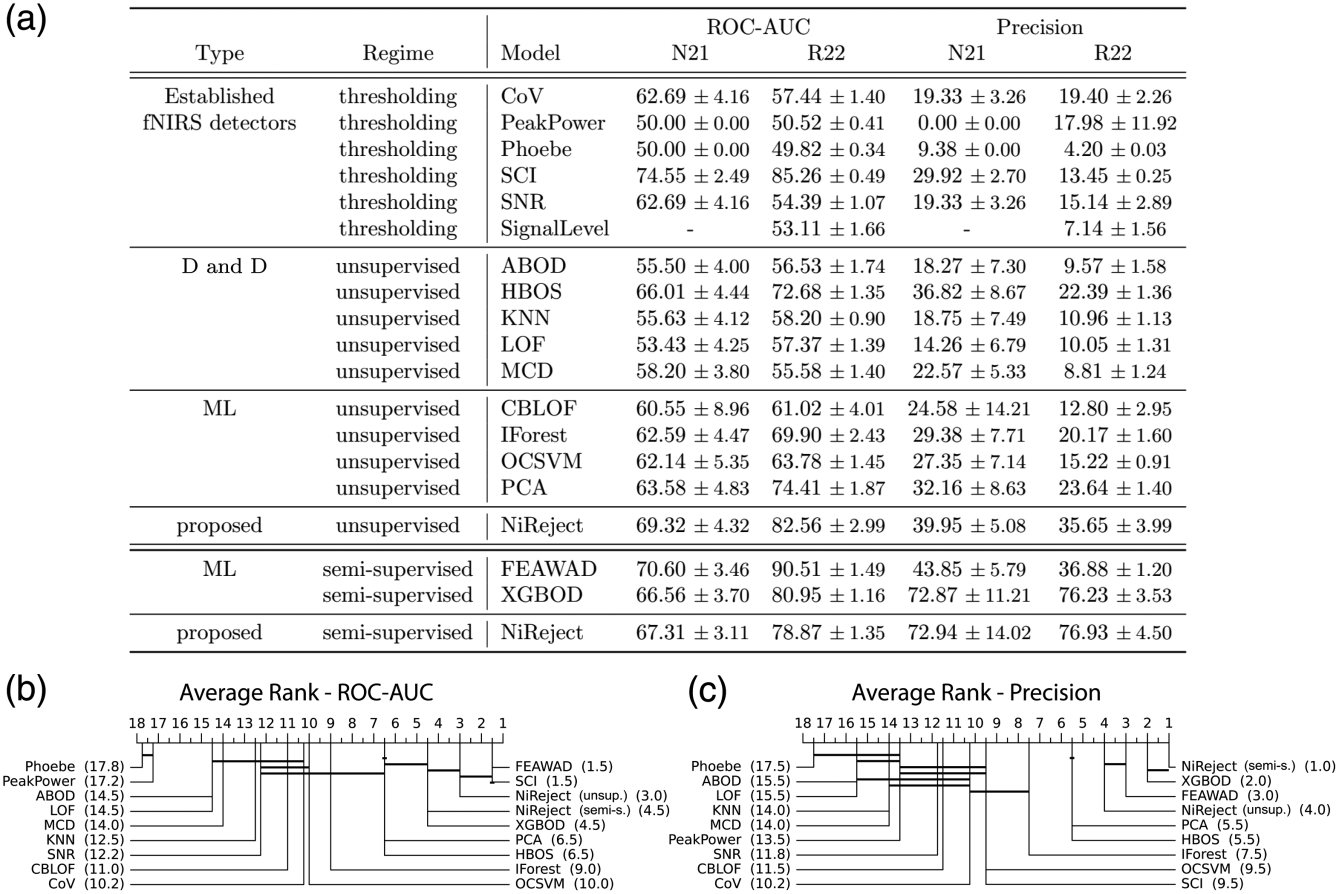
Detection performance across two real-world fNIRS datasets. (a) The mean and standard deviation of 10 repeated evaluation runs per dataset and performance metric (multiplied by 100). (b), (c) The Bayesian difference diagram, including the average detector rank across datasets in brackets (lower rank indicates better performance) and the results of the Bayesian test using horizontal lines. Thick horizontal lines connect groups of detection methods with no statistical evidence of practically relevant performance differences (CI = 90%, ROPE=[−1.0,1.0] multiplied by 100).

As depicted in Figs. 4(a) and 4(b), the SCI, a thresholding-based approach, and semi-supervised FEAWAD showed evidence for superior ROC-AUC, sharing rank 1.5 [Fig. 4(b)]. By contrast, NiReject substantially outperformed all unsupervised methods in their precision (precision rank 4.0). The higher ROC-AUC of SCI results from a high proportion of correctly detected bad channels (true positives), but this comes at the cost of increased false positives and thereby lower precision, making the SCI less practically feasible. It should be noted that Phoebe detected most channels as bad and peak power tended to detect none (see also Fig. S6 in the Supplementary Material). The signal level was not considered for N21 as it was used in the manual rating procedure of N21 (see Sec. 2.2). On R22, the signal level showed a low performance in precision and ROC-AUC. Semi-supervised methods outperformed unsupervised methods in precision [see Fig. 4(c)]. Semi-supervised NiReject outperformed all other detectors in precision with a caveat. Although the Bayesian difference plot indicates a higher rank (1.0) for NiReject compared with XGBOD (2.0), the performance difference was not of practical relevance. No strong evidence was found for improved precision of the semi-supervised detector FEAWAD (rank 3.0) compared with the unsupervised NiReject implementation (rank 4.0). Additional performance metrics can be found in Sec. S4.3 in the Supplementary Material.

Furthermore, we assessed the runtime required for training (train time) and for detection (inference time) during Q1 (see Sec. S4.2 in the Supplementary Material). Comparing the runtime on the entire train and test set showed that HBOS [$\mu_{\text{train}}$ (N21, R22): 0.18 s, 0.32 s; $\mu_{\text{inference}}$ (N21, R22): <0.01  s, 0.06 s] exhibited the best runtime. Unsupervised NiReject [$\mu_{\text{train}}$ (N21, R22): 0.27 s, 1.15 s; $\mu_{\text{inference}}$ (N21, R22): 1.08 s, 3.57 s] was among the four fastest detectors. For semi-supervised detectors, semi-supervised NiReject [$\mu_{\text{train}}$ (N21, R22): 14.39 s,: 44.40 s; $\mu_{\text{inference}}$ (N21, R22): 0.66 s, R22: 3.00 s] substantially outperformed the next best detector, XGBOD [$\mu_{\text{train}}$ (N21, R22): 109.32 s, 946.85 s; $\mu_{\text{inference}}$ (N21, R22): 21.01 s, 392.49 s].

### 3.3 Q2: Detection Behavior under Variations of Bad Channel Phenomena

**Fig. 5:**
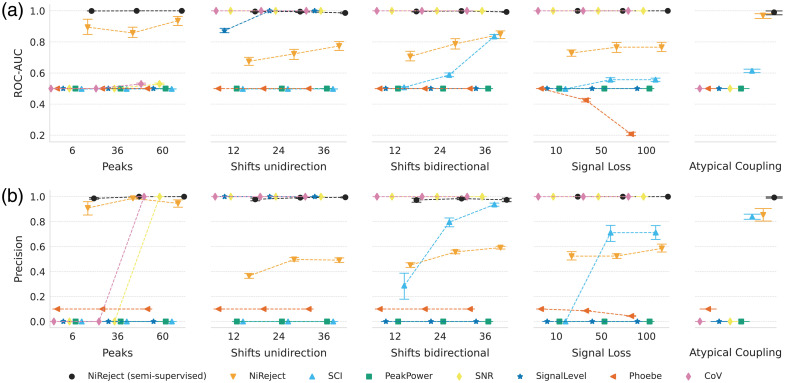
Performance profiles across bad channel phenomena. The figure shows (a) the ROC-AUC and (b) precision of color-coded detectors on simulated datasets (S1–S13). The Y-axis depicts the performance, and the X-axis encodes the different intensities for each bad channel phenomenon. Error bars represent the standard errors. Only unsupervised NiReject showed sensitivity to all phenomena.

To investigate the detectors’ performance capacities under varying bad channel phenomena, we employed synthetic datasets with either spikes, shifts, signal loss, or atypical physiological oscillations across chromophores, as described in Sec. 2.6.5. For both ROC-AUC and precision, the results suggest that established thresholding-based approaches, such as CoV, are good under certain conditions (here shifts) but fail for other phenomena, such as atypical physiological oscillations (see Fig. 5). Among this family of detectors, SCI and CoV showed sensitivity to three out of five types of phenomena. Importantly, only NiReject was sensitive to all phenomena, which might contribute to its superior performance on real-world datasets (Sec 3.2). Neither unsupervised NiReject nor the established domain-specific metrics performed best in all scenarios. Additional results, including all other detectors, can be found in Sec. S5 in the Supplementary Material.

### 3.4 Q3: Detection Behavior Under Variations of Dataset Characteristics

To guide the interpretation of detection results and explore the practical feasibility of detection methods, we assessed their performance profiles under varying contamination rates, annotation errors, and the quantity of available expert ratings in the real-world datasets (as described in Sec 2.6.6).

#### 3.4.1 Impact of varying contamination rates

Figure 5(a) depicts the ROC-AUC and precision for four detectors (SCI, XGBOD, semi-supervised NiReject, and unsupervised NiReject) that demonstrated promising performance in their respective class on real-world data (see Sec 3.2). The SCI showed a constant ROC-AUC and uptrend in precision with an increasing contamination rate and low precision at low contamination rates. This behavior can be expected because, generally, thresholding-based detectors do not depend on the contamination rate. The increase in precision seems to indicate otherwise but results from the fact that almost all bad channels and a significant proportion of signals with acceptable signal quality surpassed the SCI threshold in R22 (see Fig. 4). Consequently, an increased contamination rate led to a proportional increase in the number of true positives, whereas false positives and false negatives remained relatively constant.

Among these detectors, the semi-supervised implementation of NiReject was the only approach that scaled in its performance with an increasing contamination rate. By contrast, XGBOD demonstrated a strong degradation in ROC-AUC from c>5% onward. XGBOD’s precision increased until c=10 but then decreased with a highly fluctuating detection performance. A difference between XGBOD and NiReject is that XGBOD ensembles a latent representation of various unsupervised detectors that are highly susceptible to an increasing contamination rate and duplicate data (e.g., HBOS in Sec. S6.1 in the Supplementary Material). Consequently, XGBOD’s number of true positives does not increase with higher contamination rates, and its detection performance decreases, showing high variance. As the contamination rate is *a priori* unknown in real-world datasets, these results suggest that training on partial rating information using NiReject may provide the most reliable detection of bad channels. For results of all detectors and additional performance metrics, see Sec. S6.1 in the Supplementary Material.

#### 3.4.2 Varying available rating information

**Fig. 6:**
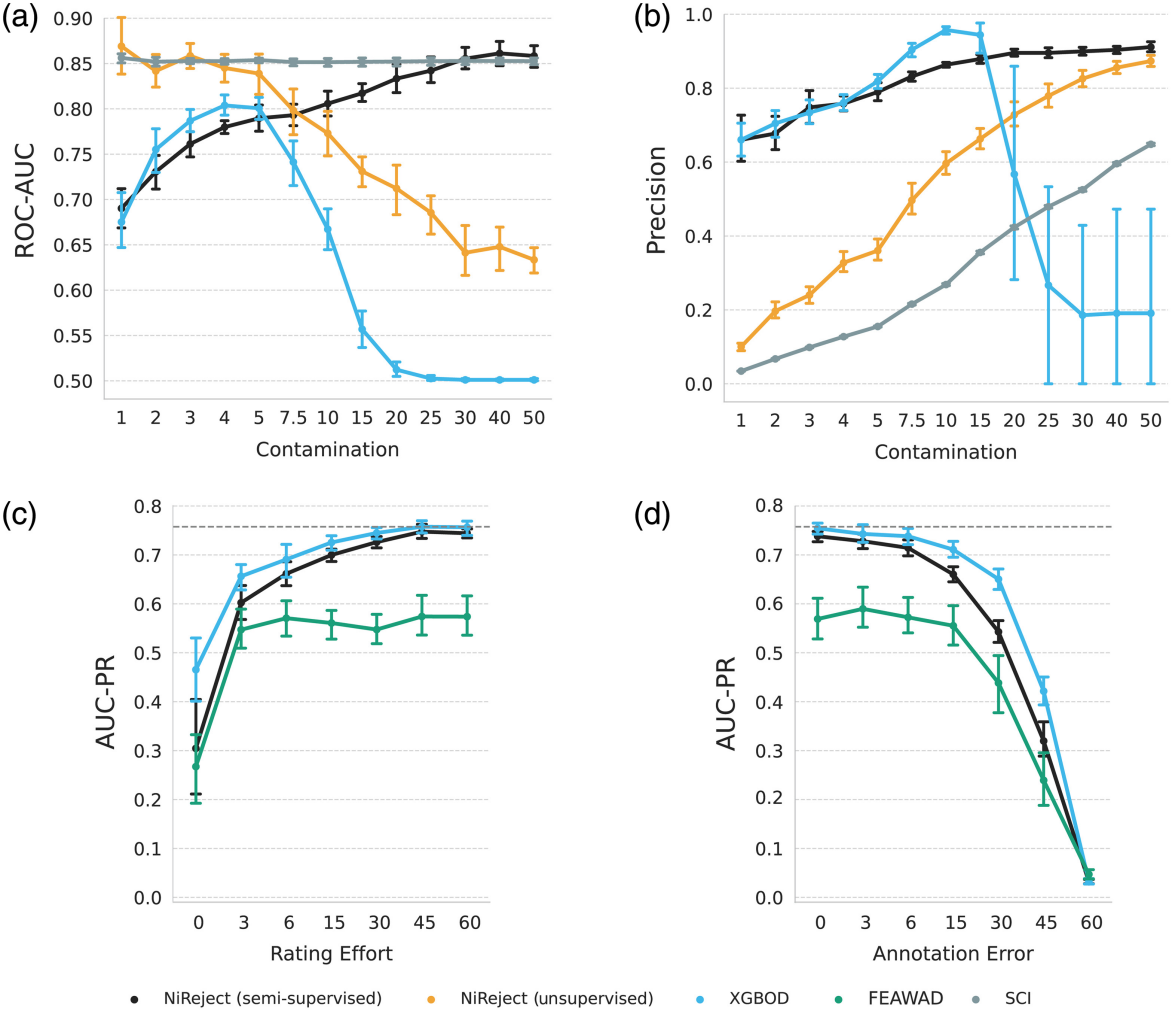
Performance evaluation across varying dataset characteristics. The figure consists of four line plots with error bars representing the standard errors. The detection performance of (a) ROC-AUC and (b) precision is depicted across varying contamination rates. Semi-supervised NiReject showed performant and robust behavior that scales with an increasing contamination rate. (c) To determine the critical number of rated bad channels for semi-supervised detectors, the X-axis encodes the ratio of rated bad channels to all bad channels in the dataset (γ), and the Y-axis shows the AUC-PR. FEAWAD reached its performance maxima with γ≥6%, and both NiReject and XGBOD approximately reached an asymptotic behavior with γ≥30% rated bad channels. (d) Depicts the AUC-PR across varying rating errors. All semi-supervised detectors showed a strong performance drop if more than 15% of rated bad channels were labeled incorrectly.

Both semi-supervised NiReject and XGBOD showed asymptotic performance behavior for γ≥30%, as depicted in Fig. 6(c). By contrast, FEAWAD required only 3%<γ<6%; however, it did not come close to the AUC-PR of semi-supervised NiReject and XGBOD. Further performance metrics can be found in Sec. S6.2 in the Supplementary Material.

#### 3.4.3 Robustness against annotation errors

As depicted in Fig. 6(d), all semi-supervised detectors demonstrated resilience to incorrectly rated bad channels of up to 15% in the dataset with overall higher ROC-AUC and precision for semi-supervised NiReject and XGBOD. Additional performance metrics on annotation errors can be found in Sec. S6.2 in the Supplementary Material.

### 3.5 Q4: A NiReject Hybrid-Detection System

In a semi-supervised regime, signals to be rated are typically selected randomly in practice. In cases of low contamination rates, this approach may result in only a few labeled nuances of bad channels, thereby deteriorating the detector’s performance or leading to a high rating effort. Thus, we assessed whether hybrid NiReject can address this challenge while outperforming unsupervised NiReject (Sec. 2.6.7).

**Fig. 7:**
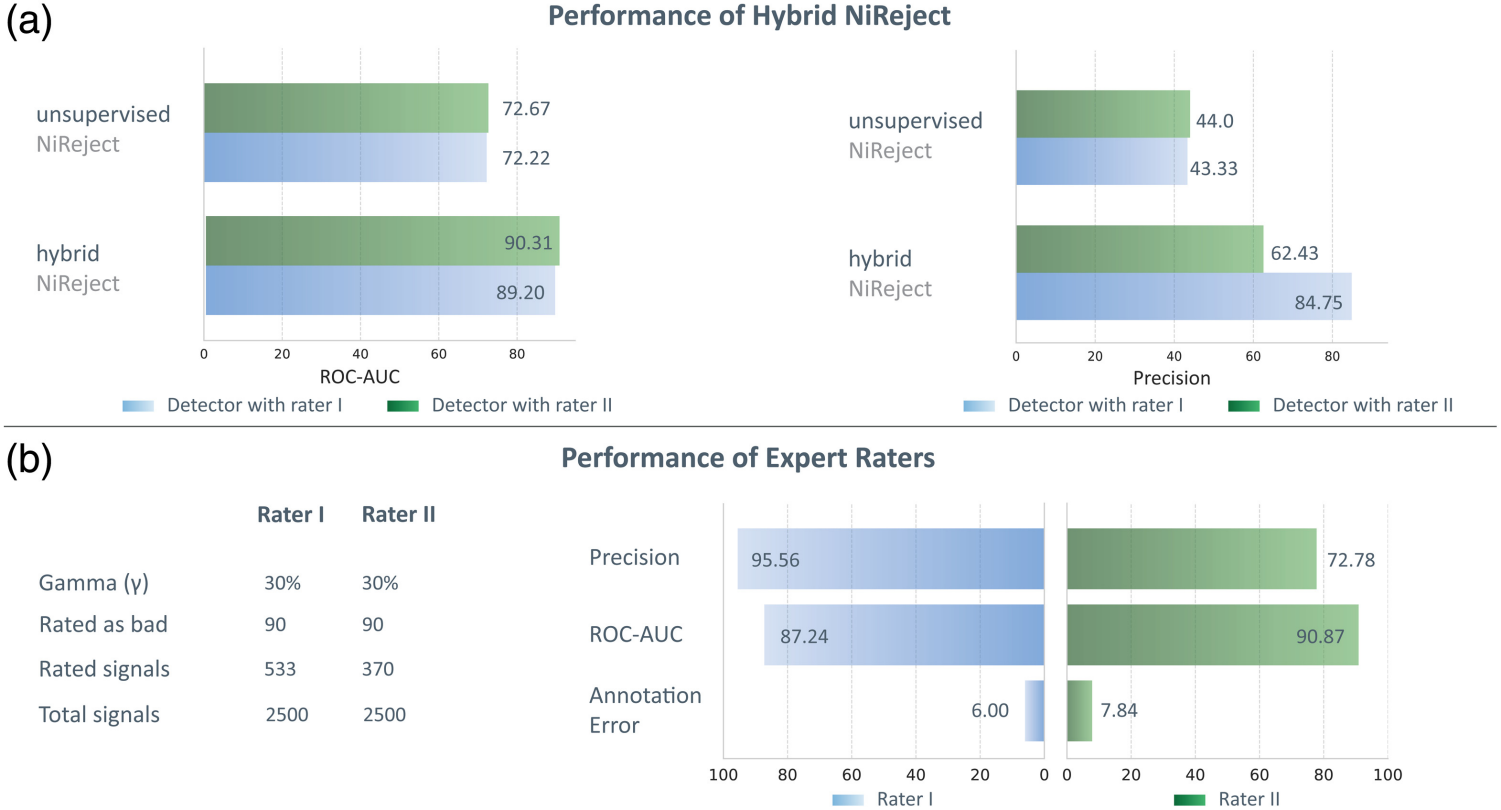
Performance evaluation of a hybrid detection system and expert ratings using synthetic data consisting of known acceptable and bad channels. (a) Shows an increase in precision and ROC-AUC of hybrid NiReject utilizing a human feedback loop compared with unsupervised NiReject. For each detector, two instances, either based on sampling involving rater I (blue) or rater II (green), are depicted. (b) Shows the rating performance of both raters. Performance to detect synthetically generated bad channels was higher in manual expert ratings compared with both hybrid and unsupervised detectors and varied between raters.

Initially, the detector assumed that 7% of the channels may be bad thereby underestimating the true contamination rate (c=30%). In the human feedback loop of the first detector instance, the expert reviewed 533 (21.32%) signals, out of which 90 (true positives: 86) were rated as bad channels. In the second detector instance, another expert reviewed 370 (14.8%) signals and assumed that 90 (true positives: 70) of these channels were bad. Precision and ROC-AUC were both high for the two raters [Fig. 7(b)]. Further details can be found in Sec. S7 in the Supplementary Material. In line with results on real-world datasets (see Sec. 3.2), our findings suggest that hybrid NiReject showed a performance increase compared with its unsupervised implementation [Fig. 7(a)].

Of note, however, is that the precision of the expert raters outperformed both model variants. Based on the results of this study, we qualitatively illustrated in Fig. 7(c) how such a hybrid system is uniquely positioned compared with other detection methods.

## 4 Discussion

### 4.1 Machine Learning-Based Detectors Can Enable Reliable, Automated Signal Quality Control for fNIRS

**Fig. 8:**
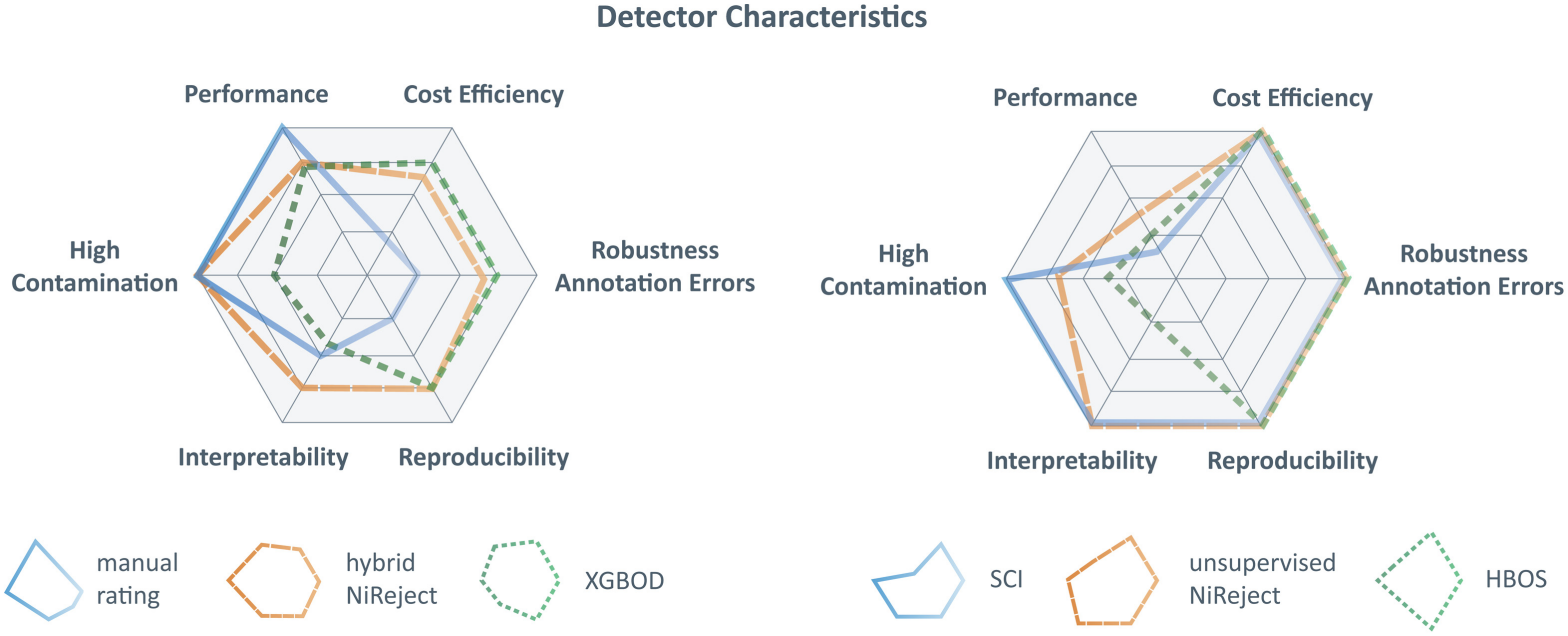
Overview of detector characteristics: This figure illustrates the characteristics of the best-performing detection methods (left) and competitive detection methods that demand low rating effort (right). The radar chart demonstrates that detectors have different profiles, which may be favored in different situations. As the distance from the center of the radar plot increases, the characteristic becomes more pronounced. Performance: Higher values mean that a detector achieves a higher detection precision. Cost efficiency: Higher values mean that fewer ratings are required for the same or better performance. Robustness against annotation errors: Higher values mean that the performance of a detector is robust against a higher percentage of rating errors before it declines. Reproducibility: Higher values mean that it is more likely to obtain the same detection results on the same dataset when detection is repeated. Interpretability: Higher values mean that the detection results can be interpreted more easily. High contamination: Higher values mean that the performance of the detector remains stable for datasets with a higher percentage of bad channels.

Although we have shown here that the detection of bad channels can influence subsequent analysis and potentially lead to incorrect conclusions, we also observed that many recent studies do not report any bad channel detection (Sec. 3.1). Studies that applied bad channel detection often relied on visual inspection. By asking experts to rate simulated fNIRS signals of acceptable and poor quality, we showed that this is indeed a viable approach that can provide high precision (Sec. 3.5). However, visual inspection also comes with several challenges, such as lower reproducibility (Fig. 8).

#### 4.1.1 Q1: Performance on real-world data

In a comprehensive evaluation based on two independently rated real-world datasets, we demonstrated that unsupervised and particularly semi-supervised machine learning-based detectors, e.g., semi-supervised NiReject (precision rank: 1.0) and XGBOD (precision rank: 2.0), exhibit strong evidence of increased detection performance compared with established thresholding-based detectors (Q1, Sec. 3.2). The SCI showed respectable performance (precision rank: 9.5) and outperformed several domain-agnostic detectors, including some unsupervised machine learning detectors. This makes the SCI the most reliable candidate among established thresholding-based approaches in the current datasets. However, its high ROC-AUC was associated with a high number of false positives. By contrast, HBOS, a classical D&D-based detector, demonstrated superior precision (precision rank 5.5) but a lower ROC-AUC compared with the SCI (Fig. S5 in the Supplementary Material). In line with Ref. 18, the CoV showed a higher similarity to manual ratings than Phoebe. Unsupervised NiReject (precision rank: 4.0) exhibited the best detection behavior across unsupervised and thresholding-based detectors, consistently achieving higher precision close to the semi-supervised method FEAWAD (precision rank: 3.0, see Fig. 4). One reason for this performance advantage results from NiReject’s lower number of false positives compared with other domain-agnostic detectors. NiReject prevents the erroneous detection of exceptionally good signals by confining its detection to a specific tail either automatically or based on prior knowledge. For example, in NiReject, only low and not high SNR values of input features contribute to identifying a bad channel. The resulting precision advancements reduce the number of unnecessary pruned signals and thereby can prevent decreasing statistical power of subsequent analyses (see also Ref. 87). These algorithmic components are also used in NiReject to decrease the computational complexity, resulting in efficient implementations of unsupervised and semi-supervised NiReject that perform training and detection in a few seconds on the larger R22 dataset (N=5922) and in milliseconds for 100 signals (details in Table S7 and Fig. S4 in the Supplementary Material). Although the runtime is less critical for ex-post bad channel detection, this should be factored in for the detector choice in future online applications, such as brain-computer-interfaces.

Of note is also that we observed some variability in the detectors’ performances between N21 and R22, which could be attributed to differences in raters, dataset characteristics, and experimental settings, e.g., differences in age groups (N21: children and adults; R22: adolescents and adults), the use of chin rests (in R22), dataset sizes, probe settings, and different fNIRS devices from different vendors. As a result, detector precision varied between datasets; for example, unsupervised NiReject (σ=0.030) and semi-supervised NiReject (σ=0.024) showed relatively low variability, whereas SCI showed higher variability (σ=0.117). In addition, within-dataset variability was significantly greater in N21, which is to be expected with a smaller sample size compared with R22 (see also Sec. S4.2 in the Supplementary Material). This underscores the importance of cross-dataset comparisons, which are currently lacking in the field.

#### 4.1.2 Q2: Variations of bad channel phenomena

To disentangle the reasons for performance differences between detectors, we next investigated the detectors under varying bad channel phenomena (Q2, Sec. 3.3). The results showed that established thresholding-based approaches might be sensitive to certain phenomena but fail to perform effectively on others (Fig. 5). These thresholding-based detectors are typically univariate and constrained by fixed thresholds, relying heavily on a close alignment between idealized bad channel phenomena and the characteristics of the actual data. This not only explains the lower performance of thresholding-based detectors on real-world datasets but also sheds light on settings in which a given detector might be less feasible. For instance, the SCI exhibited the best performance among established detectors in experiment Q1 and proved effective in capturing uncoupled wavelength changes, such as those occurring when the cap is displaced. However, the SCI did not perform reliably in signals with multiple spikes as a spike can appear simultaneously in signals of both wavelengths and consequently can result in a high SCI. For the same reason, unidirectional shifts were not detected, but bidirectional shifts, which can lead to opposing changes in both wavelengths, were. The CoV, which assesses the signal variability for each wavelength separately, is consequently more sensitive to these phenomena but requires a frequent occurrence of spikes for the overall signal variability to be affected. Moreover, the CoV criterion additionally considers the difference between the CoVs of each wavelength, but this does not necessarily capture coupling in cardiac-related frequencies. It only indicates whether one signal shows more variability than the other. As expected, due to their related definitions, the SNR and CoV demonstrated similar behavior. The signal level was only sensitive to unidirectional shifts, which is plausible because unidirectional shifts more strongly affect the amplitude in one direction. Phoebe and peak power, which is a component of Phoebe, showed no reliable detection. Future studies may investigate whether this is related to the default thresholds as it is expected that Phoebe can, to some degree, detect atypical couplings. This illustrates that the interplay between multiple thresholds in rule-based detectors might not be trivial. By contrast, NiReject and other domain-agnostic detectors demonstrated high precision across all phenomena because they operate on a multivariate feature space Z. Yet, it is important to note that no single detector outperformed all others in every phenomenon. Detectors specialized in a particular phenomenon (e.g., SCI) tended to excel in their respective niche, surpassing more generalized detectors. This supports the integration of complementary metrics, such as SCI and CoV, as features in D&D and machine learning-based detection methods. This is also in line with results from anomaly detection, which suggest that, by utilizing specific information about anomaly types (as measured by the SCI), performance improvements in detection methods can be achieved (see Ref. 36).

#### 4.1.3 Q3: Varying contamination rates

To further investigate the reliability of detectors under different dataset characteristics, we performed the experiments Q3 (Sec. 3.4). Specifically, the contamination may vary between more standardized and naturalistic tasks, involving participant movement, different populations, such as developmental and clinical cohorts and devices. Although a higher contamination rate does not affect thresholding-based detectors (see Sec. 2.6.6), unsupervised machine learning-based detectors tend to assume imbalanced settings in which bad channels represent the minority. Consequently, they are more susceptible to increased contamination rates (see Fig. S9 in the Supplementary Material) and showed to be sensitive to duplicate aberrant data (see Ref. 36). One might assume that this does not hold for semi-supervised detectors as they consider rating information. However, this only holds for semi-supervised NiReject and for the precision of FEAWAD but not for XGBOD. A notable difference between XGBOD and NiReject is that XGBOD ensembles various unsupervised detectors that are highly sensitive to increasing contamination rates and duplicated data (e.g., HBOS, see Fig. 6 and Fig. S9 in the Supplementary Material). Consequently, at higher contamination rates, XGBOD’s precision decreases with high variance, whereas the number of true positives stagnates and false negatives increases [Figs. 6(a) and 6(b) and Fig. S9 in the Supplementary Material]. Thus, the consideration of human expert ratings and the careful choice of a robust detector seems to be even more important in settings with high contamination rates. Because the contamination rate is typically unknown in practice, training NiReject on partial rating information from human experts may offer reliable detection of bad channels across varying contamination rates. Further, the SCI, a thresholding-based approach, appears to be a suitable alternative that offers a more robust detection than most unsupervised machine learning-based detectors for high contamination rates (Fig. 8).

#### 4.1.4 Q4: Varying available rating information

Because semi-supervised methods depend on partially available rating data, we varied the amount of available rating information. FEAWAD’s performance plateaus at γ=6% and the asymptotic behavior of XBOD and semi-supervised NiReject starting at γ=30% suggest that semi-supervised approaches demand only some rating information. FEAWAD’s lower demand can result from its autoencoder-based architecture, which only “recalibrates” the encoder by a few bad channels. Nevertheless, in a more conservative setting, a higher γ might be desirable, whereas a lower γ may be sufficient in less conservative settings.

#### 4.1.5 Q5: Robustness against annotation errors

All semi-supervised detectors remained relatively robust against annotation errors of raters unless more than 15% of the data was rated incorrectly. This suggests that semi-supervised detectors can maintain a robust performance up to a certain level of annotation errors before these errors begin to bias the detector toward a strongly reduced performance. This is in line with findings in anomaly detection, which show that semi-supervised and supervised detectors are robust against minor annotation errors (although with lower robustness for supervised).^36^ However, of note in this study is that other semi-supervised detectors (e.g., GANomaly) were more robust than FEAWAD and XGBOT. Nevertheless, the importance of accurate ratings for precise detection of semi-supervised, hybrid, and supervised models should not be underestimated even when less than 15% of annotation errors occur (see Sec. 3.5). Thus, to ensure a high rating quality, it may be advisable to train human raters using simulations or available datasets and, in the case of multiple raters, to measure their inter-rater variability. Further, providing clear rating instructions ideally with examples and counterexamples can guide decisions and enhance rating performance (see also Ref. 17). However, overall, the resilience of semi-supervised detectors to annotation errors can be an advantage over visual inspection alone.^36^

#### 4.1.6 Q6: Assessment of the NiReject hybrid-detection system

Although these findings highlight the efficiency and robustness of semi-supervised detectors, it remains open for practitioners to decide which signals should be rated first. Because bad channels are typically a minority, a substantial rating effort may be required before reaching a suitable percentage of bad channels, which could offset the benefits of semi-supervised learning. Results of Sec. 3.5 suggest that the proposed hybrid NiReject efficiently leveraged expert feedback in a human feedback loop, yielding a substantial performance increase compared with pure unsupervised detection while mitigating the challenges and high manual rating effort of supervised machine-learning methods (proportion of rated data: 14.8% to 21.32% in hybrid NiReject compared with 75% in supervised detection^31^). Furthermore, from Eq. (4) (Sec. 2.5.1), it follows that the degree of feature d to which the signal i is expected to be a bad channel can be directly obtained. Thereby, NiReject models provide a model intrinsic interpretability guiding practitioners to understand the results [Fig. 2(d)]. Consequently, this hybrid system enables a relatively interpretable and reproducible bad channel detection while remaining performant and cost-efficient also in high contamination cases (Fig. 8).

### 4.2 Transparent Reporting of Bad Channels

Given the sparse reporting and the scarcity of studies on fNIRS bad channel detection, the question of how to adequately report bad channels remains largely elusive. When bad channel detection is reported, most studies tend to provide only basic information (parameters/thresholds: 20%, number of rejected channels: 7%, number of rejected subjects: 3%, see Sec. 3.1). Future research should aim to provide at least a basic description of bad channel detection in the main text, including the toolbox used, detection method, parameters, contamination rate, number of rejected signals, and rejected subjects. The authors of Ref. 88 additionally visualized which long-distance channels of which subjects were excluded. This can yield valuable information on the channels that are of consistently poor quality, on subjects with particularly low data quality, and on systematic quality differences between groups/conditions that might require additional considerations in downstream analysis, as in Refs. 89 and 90.

**Fig. 9:**
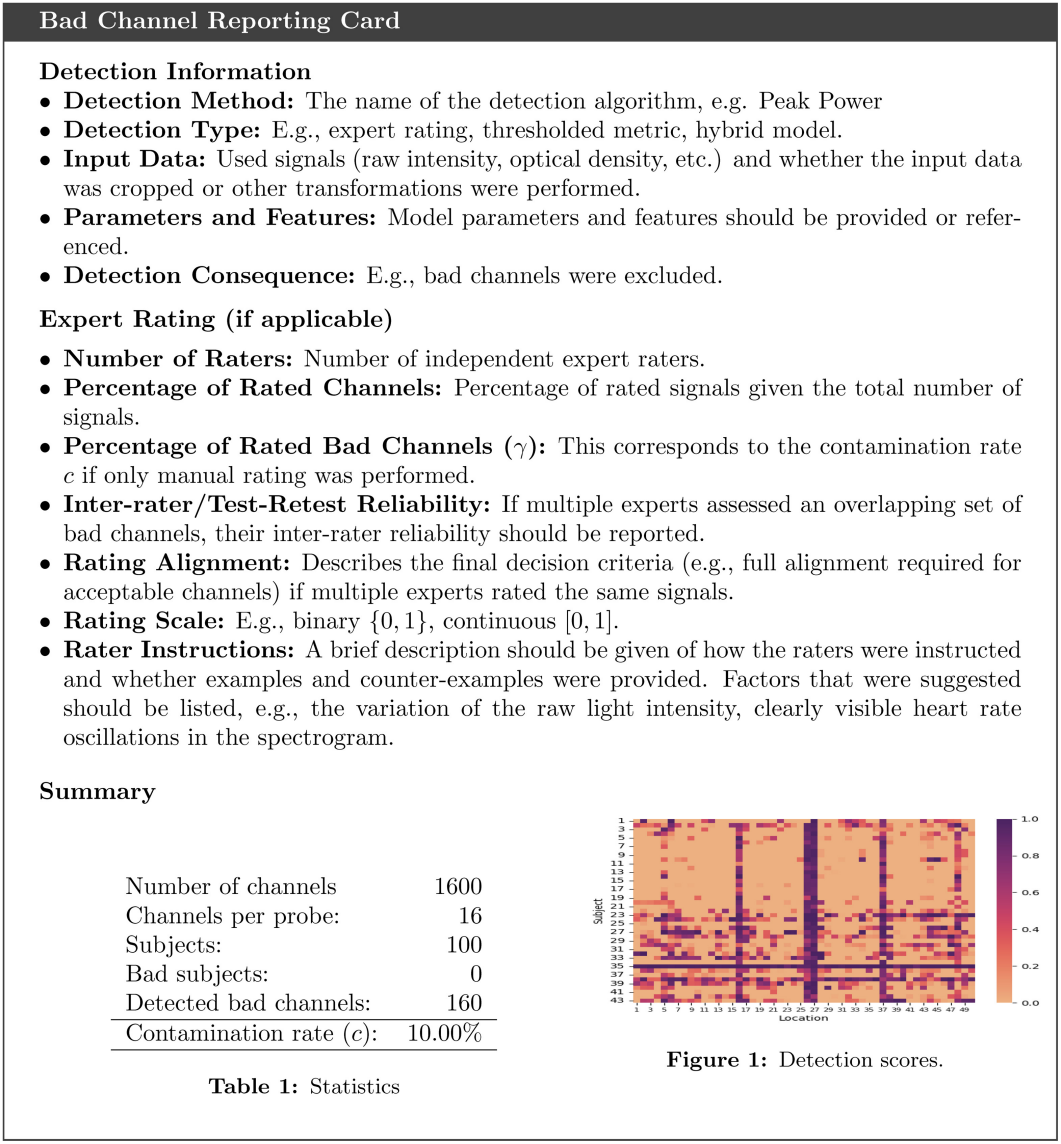
Bad channel reporting card. The figure displays the template for the bad channel reporting card that contains basic information about the detection method, statistics, and detection scores across channels and subjects.

To enhance the transparency and reproducibility of fNIRS studies, we suggest a systematic bad channel reporting, as provided in Fig. 9. This reporting card is designed to provide details on the employed detection methods and expert rating (if applicable), as well as a summary of the detection details. This information not only may contribute to a more transparent reporting but also can guide researchers in their analysis decisions, e.g., which signal and subjects to exclude from downstream analysis. A template of the reporting card is provided alongside the publication (see Sec. Code, Data, and Materials Availability) and can be used alongside previously published guidelines for transparent reporting of fNIRS studies.^15^

### 4.3 Limitations and Future Directions

Based on the current state of the field and the systematic assessment provided in this study, we note several limitations of the study and deduce future directions for improved signal quality control in fNIRS.

#### 4.3.1 Choice of thresholds

Here, we evaluated thresholding-based detection methods using common default parameters. It is important to note that other thresholds may lead to different results and that the best threshold parametrizations are not known in practice.

#### 4.3.2 Effects of age

Because physiology, anatomy, and behavior vary from age to age, the same preprocessing methods and parameterizations may not be equally suitable for all ages. To account for this, we extended the peak power metric by applying the physiologically parametrized power spectrum of neural signals (FOOOF).^75^ However, a systematic examination of age effects is beyond the scope of the current paper and may require larger datasets per age group of infants, children, and adolescents (Sec. S4.1 in the Supplementary Material). This could help to determine whether age-specific detection methods or adaptations are beneficial.

#### 4.3.3 Bad short-distance channels

Bad channel detection often focuses on long-distance channels as these are primarily used in subsequent analyses. A widely adopted and recommended approach to reduce the systematic influence of physiological noise is short-channel separation, i.e., using short-distance channels to regress out physiological noise from long-distance channels.^91^ Although this is a valuable approach, it is important to note that a bad short-distance channel can also impact signals of long-distance channels instead of improving their quality. To mitigate this corruption, measuring multiple short channels and rejecting bad short channels is desirable. Specifically, semi-supervised NiReject has the ability to differentiate between the detection of short- and long-distance channels due to its annotation of spatial information (Sec. 2.5.2). However, particularly for unsupervised and thresholding-based detectors a separate detection of long- and short-distance channels may be beneficial. Thus, future studies may investigate bad channel detection methods and their features, such as their thresholds, specifically for long- and short-distance bad channels.

#### 4.3.4 Multimodal bad channel detection

As some modern signal enhancement methods have been shown to benefit from multimodal data (see Sec. 1), future studies could aim to integrate additional data types, such as video or accelerator data, into detection models. This can help, for example, to better detect movement-related artifacts or displacements in optodes and thus improve the detection of bad channels.

#### 4.3.5 Online bad channel detection

It is also worth noting that NiReject and several of the other detection methods assessed in our study are primarily designed for an ex-post application after data collection. Some of the detectors, such as HBOS and NiReject, exhibited an efficient runtime that could be sufficient for online detection, e.g., in online neurofeedback analysis (e.g., Refs. 92 and 93) or brain-computer interfaces. Future studies may develop and empirically assess implementations that enable online/batch processing of bad channels.

### 4.4 Conclusion

With an increasing number of channels per device, larger cohorts, and the pressing need for more standardized processing procedures, thresholding-based detectors, such as the CoV or SCI, are increasingly employed in fNIRS. However, despite their advantages, unsupervised and semi-supervised detectors have not yet been used. In the present study, we performed a comprehensive and systematic assessment across a landscape of 19 established, unsupervised, and semi-supervised approaches for detecting bad channels in fNIRS. We provided an overview of the current state of signal quality control, demonstrated the pivotal role of bad channel detection in subsequent analysis, and investigated how the nuanced behavior of detection methods determines their practicability and reliability. The probed key characteristics revealed how detectors differ in their performance capacities, cost-efficiency, and robustness under varying conditions. When fully automated detection without any manual rating is desired, unsupervised NiReject demonstrated overall a more precise detection than all other approaches. Generally, machine learning detectors that leverage partially rated data, particularly NiReject, achieved superior detection performance compared with established thresholding-based and unsupervised detectors. Among these methods, the semi-supervised NiReject stood out due to not only the best runtime but also providing the most robust performance under challenging settings in which a higher number of bad channels can be expected and, as the other semi-supervised detectors, was relatively robust against annotation error. In addition, hybrid NiReject extends semi-supervised methods by suggesting which signals to rate. This may come with a high precision but at a lower rating effort compared with semi-supervised detection. Our work may motivate future artificial intelligence-based developments in offline and online data quality control and correction, applicable across a wide range of populations and environments to ensure robust and high-quality neuroimaging findings.

## Supplementary Material



## Data Availability

The data presented in this article are publicly available in the repositories listed below. Ensuring transparency and reproducibility, all experiments were conducted within a technically reproducible docker container and tracked using MlFlow. Parameters and performance metrics were stored in an artifact storage and provided along with the GitHub repository. Artifacts and benchmarking data (R22, synthetic datasets) were versioned and publicly distributed using GIN (G-Node Infrastructure). To comply with best practices from software development, we additionally implemented unit tests. Code repository: https://github.com/ChristianGerloff/nireject-publication. Artifact data repository: https://gin.g-node.org/ChristianGerloff/nireject-publication. Public datasets R22, synthetic data: https://gin.g-node.org/ChristianGerloff/nireject-benchmark. Public dataset N21: https://osf.io/wspz4/. Bad channel reporting card template: https://github.com/ChristianGerloff/badchannelcard.
