# Supplementary material for: NiReject: toward automated bad channel detection in functional near-infrared spectroscopy
Source: Neurophotonics. 2024 Nov 4;11(4):045008. doi: 10.1117/1.NPh.11.4.045008 (PMC11532795; doi:10.1117/1.NPh.11.4.045008)
Supplement: Supplementary file 1 [file NPh_011_045008_SD001.pdf]

Supplementary Material

1. Structured literature search

The structured literature search was conducted using the open-source search engine SetYouFree[84], which follows the best practices of the PRISMA guidelines for systematic reviews. Detailed search results can be found in SI Files Search Results (Excel table).

To select the 100 most cited publications in the field of fNIRS published in 2022, we utilized the Scopus database, which provides citation counts. Duplicate publications and commentaries were automatically merged based on the similarity of abstracts and titles calculated from the enhanced version of the Ratcliff-Obershelp algorithm. Subsequently, titles and abstracts were automatically screened for predefined exclusion criteria. The initial screening was checked and revised by a manual reviewer. Finally, reporting details for the 100 most cited publications were extracted from the 559 remaining studies by a manual reviewer. The main search parameters for the systematic search can be found in the following tables.

Further quality criteria that were identified include average signal level (N=2, Homer), flat line (N=2), gain / gain factor (nirsLAB, N=2) Pearson correlation of HbO and HbR (N=1), anti-correlation (N=1), q-threshold (QT-Tool, N=1), Standard deviation of optical density (N=1) and one study reported intensity and saturation as quality criteria but did not provide further specifications.

An additional unstructured search was performed ex post. Based on the cross-references of the existing supervised-machine learning detectors[31,42] two relevant studies were found[85,86] that cited and applied these detectors.

Table S1 Search parameters of structured literature search.

Parameter	Value
Databases searched	Scopus
Search string	[functional near-infrared spectroscopy] OR [fNIRS]
Publication types	Publications and conference proceedings including: methodological studies and empirical studies
Start date	2022-01-01
End date	2022-31-12
Conducted at	2023-10-26
Similarity threshold	0.9
Exclusion criteria	Non neural studies, reviews, guidelines, protocols, non-human studies, not available in English, citation count (top 100)
Extraction criteria	Detection not reported, Visual Inspection / manual, Coefficient of Variation (CoV), Scalp Coupling Index (SCI), Peak Power, Phoebe, Signal Quality Index (SQI), Signal-to-noise-ratio (SNR), Other detectors, Parameter, number of rejected signals reported, specific rejected signals reported

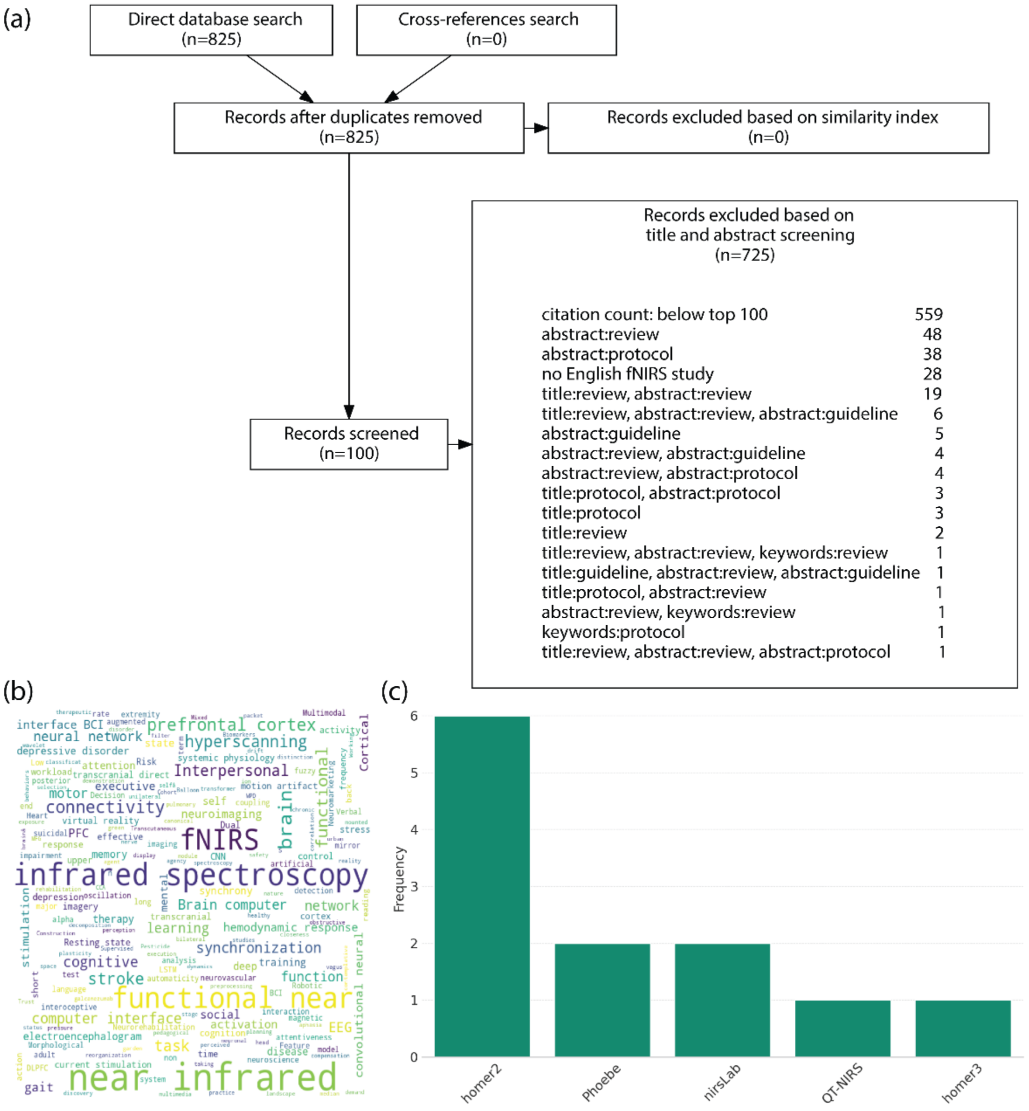

Fig. S1 Search Details. (a) shows the flow diagram showing the step-by-step process of the structured literature search following PRISMA guidelines from initial identification of studies through to final inclusion in the review. The "Records excluded based on title and abstract screening" box provides a detailed breakdown of the reasons for excluding records according to the defined exclusion criteria. Where applicable, we report location:criteria, e.g., 48 studies were excluded because the word "review" was identified in the abstract (abstract:review… 48). (b) shows the word cloud of the structured search, including prominent keywords. The size of each keyword corresponds to its frequency of occurrence within the dataset. Common themes emerge as larger, bolder words. (c) depicts the frequency information of toolboxes employed for bad channel detection. In 12 out of the 32 studies that applied bad channel detection, one of the depicted toolboxes was reported as being used for detection; in the other 20 studies, no toolbox was reported for detection.

1.1 Detection methods in fNRIS signal quality control

Detection methods found in the structured literature search are depicted and described in table S2. Additional methods that were not found in the systematic literature search are described in table S3. It should be noted that generally some of the methods (e.g., CoV, peak power, SCI, SNR) may also be calculated based on the average or median value of the metric across time-windows of a signal. Common parametrizations of the most dominant detection methods can be found in Table S4.

Table S2 Methods for bad channel detection in fNIRS**.**

Detection method	Regime	Integration	Studies	Description
Visual inspection	manual		[26,94–104]	Visual inspection of fNIRS signals is performed after data acquisition based on the judgment of a human expert. Human expert raters may inspect signal characteristics such as signal variability and evidence of cardiac related oscillations (see [27]).
Anti-correlation	threshold	*MNE-NIRS (implemented for signal enhancement)*	[26]	The criterion is based on the coupling of HbO and HbR [105]. As an increase in the concentration of HbO should be accompanied by a decrease in HbR, both chromophores should be negatively but not perfectly negatively correlated. This criterion was originally employed for signal enhancement to increase the negative correlation between signals of both wavelengths.
Coefficient of variation (CoV)	threshold	LIONirs, nirsLAB	[26,99,106–111]	The CoV is a widely applied, scale-invariant metric [25,52] with a reciprocal relation to SNR ($SNR = 1/\mathrm{CoV}$). It is calculated as the ratio between standard deviation and mean amplitude. Hence, an increasing CoV indicates a decreasing signal quality. A threshold is usually applied to the CoVs of both wavelengths and often additionally to their difference (e.g., [25,27]). By this, the metric captures unexpected variability within the photo-detected signals and between both wavelengths. It is typically calculated based on the raw light intensity, independently for each channel and subject but it can also be calculated across multiple signals (e.g., [53]).
Flat line	threshold	-	[26,111]	This quality criterion assumes a signal is bad if the raw light intensity or optical density exhibits a constant or near-constant amplitude over a sustained period of time. A flat line may be caused by, for example, poor optode-scalp coupling or instrument malfunction. Its decision function is usually based on a threshold specifying a critical duration of flat lines[27] or their proportion with respect to the total signal length[111].
Gain factor	threshold	nirsLAB	[99,107]	The gain factor describes how the photocurrent produced by the light signal is amplified, and increases with deceasing SNR.[54]
Peak Power	threshold	MNE-NIRS, NIRS brain AnalyzIR toolbox, Phoebe, QT-NIRS	[112,113]	Peak power (also termed, ”spectral peak power”; [23]) judges bad channels based on the spectral power of the cross-correlated attenuation. Since cardiac signals can be modeled with two sinusoidal waves, the peak power mainly addresses spikes or baseline shifts that occur concurrently in both wavelengths in a predefined cardiac-related frequency spectrum.
Placing Headgear Optodes Efficiently Before	threshold	Phoebe,	[112,113]	Is the name of a bad channel criterion composed of SCI and peak power. It assumes a bad channel if either the SCI or peak power criterion is fulfilled. Thereby, it aims to prevent bias from unreasonably high SCI values, which can result from motion artifacts leading to synchronous peaks across wavelengths.

Experimentation (Phoebe)		NIRS brain AnalyzIR toolbox<br><br>QT-NIRS,		
q-threshold	threshold	QT-NIRS NIRS brain AnalyzIR	[113]	Similar to Phoebe, this criterion consists of the SCI and peak power. However, it is based on the frequency at which either the SCI or peak power criterium detect a bad channel in a windowed signal.[30] If a proportion of signal segments above a certain threshold is detected by either criterion, the segment is considered bad.
Scalp coupling index (SCI)	threshold	MNE-NIRS, NIRS brain AnalyzIR toolbox, Phoebe, QT-NIRS	[112–114]	SCI[20] is defined as the zero-lag cross-correlation between both wavelengths of the raw attenuation, whereby higher values indicate better coupling. This is based on the assumption that synchronous cardiac pulsation of photo-detected signals is known to indicate a good contact between optodes and scalp.[55,56] The SCI can be derived either directly from the entire signals or from the median of windowed signals, which is less affected by signal length.
Signal level	threshold	Homer2&3, LIONirs, NIRS brain AnalyzIR toolbox	[88,115]	Signal level, or average signal level, is a quality metric calculated from mean amplitude of raw light intensity. This thresholding-based detection method rejects signals of low (e.g., [53]) or high intensity or both (e.g., [22]). Thereby it assumes that low intensity, e.g., from insufficient light penetration or scattering, and high light intensity, e.g., from ambient light sources, can affect fNIRS analysis. This metric can also be calculated from optical density measurements.
Signal-to-noise-ratio (SNR)	threshold	Homer2&3, NIRS brain AnalyzIR	[104,108,88,115–122]	The SNR has an inverse relationship with the CoV, meaning that a lower SNR implies decreasing signal quality. [22] It is often calculated by dividing the standard deviation of signals by their mean (Ref. [22]) or median (Ref. [48]). While CoV and SNR might be used interchangeably, implementations based on SNR often capture only the unexpected variability within photo-detected signals but not between signals of different wavelengths.
Spatial correlation across channels	threshold	-	[123]	The criterion assumes that acceptable signals that capture task-related changes in blood flow demonstrate a high spatial correlation to signals from the same hemisphere.[123] Therefore, an acceptable channel requires reaching a Pearson correlation above a threshold in both HbO and HbR with at least one other channel in the same hemisphere. The criterion was derived from functional connectivity analysis in fMRI.
Standard deviation	threshold	-	[124]	The standard deviation of optical density is used to reject signals with very low or high optical density.[124] This approach can differ from CoV and SNR in that it does not necessarily take the signal level into account.

Note: Rows are alphabetically ordered for each regime. Column “Integration” describes whether the detection method is implemented in one of the following popular toolboxes: Homer2&3[21], LIONirs[53], MNE-NIRS[57], NIRS brain AnalyzIR toolbox[48], nirsLAB[54], Phoebe[23], QT-NIRS[30]. Column “Studies” lists the related publications from the structured literature search in which the method was employed.

Table S3 Other methods**.**

Detection method	Regime	Description
Light-tissue coupling index	threshold	This approach calculates based on HbO the quotient of power in heart-rate related frequency and power above heart rate [24]. Thereby it describes the ratio between the magnitude of blood volume pulsation and high-frequency noise, e.g., caused by improper light-tissue coupling.
Maximal Variability Expectation	threshold	This detection method employs a probability mass function (PMF) representing the behavior of variance within the HbO signals[21]. Channels increasing the skewness of the PMF above a threshold are considered bad channels. The method assumes that the strong cardiac-respiratory coupling across fNIRS signals should lead to Gaussian distributed PMF for signals of acceptable quality.
Signal quality index (SQI)	threshold	The SQI[29] employs a fitted regression model resulting in five thresholded metrics to distinguish quality profiles of channels in three steps. First, the thresholded maximum raw light intensity, the standard deviation of the optical density per wavelength, and ratio of oxygenated and deoxygenated hemoglobin summation are used to identify a bad channel. Next, channels of acceptable quality are determined by comparing the autocorrelation of optical density of both wavelengths and thresholding the standard deviation of the difference between the autocorrelations. Channels are either assigned as bad or acceptable for a deterministic decision or are ranked from one to five based on the logarithm of the ratio between the standard deviation of HbO and HbR.
Structured noise index (SNI)	threshold	The SNI[125] is calculated across channels and participants. It measures the ratio of the variance in the complete dataset to the variance observed in an auto-regressively whitened version of the same dataset. Thereby it quantifies the ratio between structured noise from physiological processes and white noise. The SNI is implemented in the NIRS brain AnalyzIR.

Note: Besides the methods in table S3. NIRS brain AnalyzIR also offers to report a stationarity test (Kwiatkowski, Phillips, Schmidt, and Shin test) and the Anderson-Darling test as general quality indicators

Table S4 Common parameters of dection methods in fNIRS.

Detection method	Parametrization
CoV	threshold CoVs of both wavelengths: ≥ 0.1 (10%), threshold of their difference: ≥ 0.05 (5%), denominator: mean
SNR	threshold: <10, denominator: mean
SCI	threshold: < 0.5, window size 2-10 s, aggregation: median
Peak power	threshold: < 0.1, window size 2-10 s, aggregation: median
Phoebe	SCI < 0.5, peak power < 0.1, window size 2-10 s, aggregation: median
Signal level	Depends on measurement scale: <0.03, ≥ 2.5 or <3, ≥ 250

Note: The operator in the column ”parameterisation” specifies the condition for a bad channel. In accordance with common practice, a bandpass filter is applied before SCI, peak power and phoebe (type...iir, limits... [0.7 Hz, 1.5 Hz], order...4).

2. NiReject: Further mathematical description

The joint empirical cumulative distribution function along the d-th dimension of Z can be derived from factorization as follows[68]:

$$\begin{aligned}\hat{F}_{left}(z) &= \frac{1}{n}\sum_{i=1}^{n} \mathbb{1}\left\{Z_i^{(1)} \leq z\right\} \times \mathbb{1}\left\{Z_i^{(\dots)} \leq z\right\} \times \mathbb{1}\left\{Z_i^{(d)} \leq z\right\} \\ &= \prod_{j=1}^{d} \hat{F}^{(j)}\left(z^{(j)}\right) \\ &= \prod_{j=1}^{d} \mathbb{P}\left(Z_i^{(j)} \leq z\right).\end{aligned} \quad \text{(A1)}$$

The same applies to $\hat{F}_{right}(z)$. If no priors are given, we must correct for the case that outliers can have a tendency to lie in one tail or another. Consequently, the skewness coefficient, denoted by φ_j, is calculated for the j-th feature distribution:

$$\varphi_j = \frac{\frac{1}{n}\sum_{i=1}^{n}\left(z_i^{(j)} - \overline{z^{(j)}}\right)^3}{\left[\frac{1}{n-1}\sum_{i=1}^{n}\left(z_i^{(j)} - \overline{z^{(j)}}\right)^2\right]^{3/2}}, \quad \text{(A2)}$$

whereas $\varphi < 0$ suggests a distribution with longer left tail, while $\varphi \geq 0$ indicates a longer right tail as described in [68]. With an increasing number of features, the tail probability vanishes. Therefore, the left, right and skewness corrected tail probability, described by $W(Z_i)$, was calculated in negative log-space [68]:

$$W(Z_i) = -\sum_{d=1}^{j} \mathbb{1}\{\varphi_d < 0\}\log\left(\hat{F}_{\text{left}}^{(j)}\left(Z_i^{(j)}\right)\right) + \mathbb{1}\{\varphi_d \geq 0\}log\left(\hat{F}_{\text{right}}^{(j)}\left(Z_i^{(j)}\right)\right). \quad \text{(A3)}$$

In a similar way we can derive each feature and signal specific tail probability:

$$W^{(j)}\left(Z_i^{(j)}\right) = -\mathbb{1}\{\varphi_j < 0\}\log\left(\hat{F}_{\text{left}}^{(j)}\left(Z_i^{(j)}\right)\right) - \mathbb{1}\{\varphi_j \geq 0\}log\left(\hat{F}_{\text{right}}^{(j)}\left(Z_i^{(j)}\right)\right). \quad \text{(A4)}$$

3. Impact of bad channel detection: analysis details

3.1 First-level analysis example: average waveform analysis

Data ingestion and first level analysis were performed in Python, using customized versions of the "MNE-NIRS". First, we converted the raw fNIRS signal to optical density. For the analysis with bad channel detection, bad channels, which were a priori known, were excluded. To reduce motion artifacts, we applied the Temporal Derivative Distribution Repair[126] to optical density. Haemoglobin was calculated using a partial path length factor of 0.1. A bandpass filter using frequency bounds (0.01 Hz, 0.7 Hz) and transitions (0.005, 0.3) was applied. Short and long-distance channels were separated based on the optode distance (short: d < 15mm, long: 15 mm ≤ d ≤ 45 mm). Epoched task data of long-distance channels were used to calculate the average waveform across all channels of each subject. Finally, average waveforms with and without bad channel detection were plotted for a single subject. The MSEs between the average waveform from the preprocessed signals and ideal hemodynamic response function were calculated.

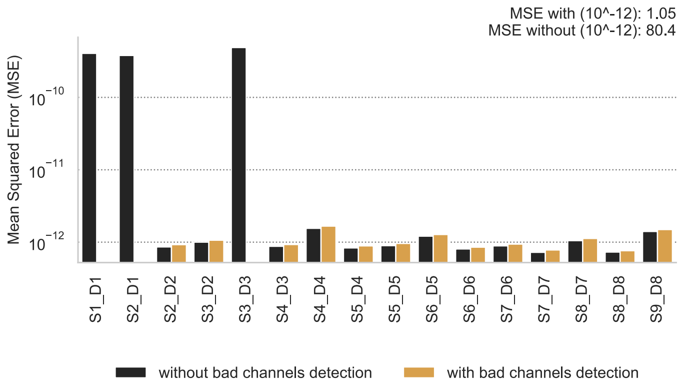

Fig. S2 Mean square error (MSE) between average waveform of single subject's preprocessed signals from long-distance-channels and ideal hemodynamic response function. The bar plot shows the MSE for each channel of the subject with ideal and without any bad channel detection. Bad channels were simulated in channel S1_D1, S2_D1 and S3_D3 and rejected during bad channel detection. Mean MSE across channels is displayed in the upper right corner. A lower MSE implies a better reconstruction of the ideal hemodynamic response function.

3.2 Group-level analysis example: "GLM analysis"

Changes in haemoglobin concentration were derived in the same fashion as in Sec. S3.1. Instead of calculating the average waveform, the response estimates per channel, denoted by Θ, were calculated from a fitted GLM. The GLM fitted an HRF basis function (Glover) based on the long channels and used short channels to regress out physiological noise. The simulated stimulus duration (2 s) was considered in the GLM. Further, we incorporated an autoregressive model with the order being four times the dataset-specific sampling frequency. For each subject, condition (task, rest) and channel estimate, results against zero were calculated. Subsequently, we utilized a linear mixed effects model [Θ ~ -1 + channel (1| subject id), data = contrast (task vs. rest) of HbO] to examine the effect of task vs. rest on response estimates. Finally, group results of the task vs. rest contrast were projected on the nearest cortical surface with and without bad channel detection.

In Table S5 the statistical results of the GLM are summarized for the analysis with and without bad channel detection. The results showed that neglecting bad channels can lead to wrong conclusions about region specific effects.

Table S5 GLM: Channel results for HbO.

Channel	P-Value With Detection	P-Value Without Detection	Significance With Detection	Significance Without Detection
S1_D1	0.0000	0.0000	***	***
S2_D1	0.3196	0.1967	None	None
S2_D2	0.0000	0.0005	***	***
S3_D2	0.0000	0.0000	***	***
S3_D3	0.0000	0.0000	***	***
S4_D3	0.0000	0.2204	***	None
S4_D4	0.0000	0.0001	***	***
S5_D4	0.0000	0.0000	***	***
S5_D5	0.0000	0.1111	***	None
S6_D5	0.0028	0.0007	**	***
S6_D6	0.0227	0.2240	*	None
S7_D6	0.0000	0.0000	***	***
S7_D7	0.0000	0.0292	***	*
S8_D7	0.0000	0.0000	***	***
S8_D8	0.0000	0.0175	***	*
S9_D8	0.0000	0.5788	***	None

Note. Red marks an underestimated effect and blue indicates an overestimated effect. * $p < .05$, ** $p < .01$, *** $p < .001$.

4. Detection performance on real-word datasets: additional results

4.1 Bad channels per age group

The number of bad channels per dataset (N21, R22) and age group (adults, children, adolescents) is depicted in Fig. S3. Of note is that the total number of channels per wavelength assessed is $N = 640$ for N21 (220 channels from 20 children and 220 channels from 20 adults) and $N = 5984$ for R22 (2992 channels from 34 children and 2992 channels from 68 adults). The datasets show an imbalance in the number of "true" bad channels across different age groups, particularly for dataset N21. Notably, the number of bad channels for adults of N21 is very low. While this is not problematic for practical applications, it would make a separate benchmarking for each of the age cohorts unreliable.

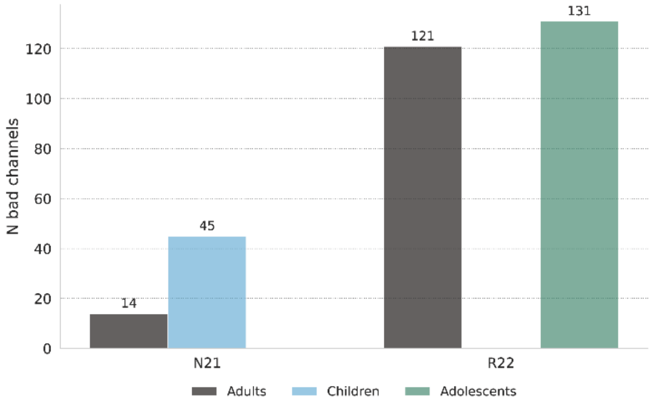

Fig. S3 Number of bad channels per age group of datasets N21 and R22.

4.2. Runtime comparison

Runtime efficiency. We assessed the runtime efficiency during the ten runs (repeated train-test splits) in experiment Q1. Table S6 and Table S7 depict the runtime for training on the training set and the inference time, measuring detection on the test set. For machine learning-based and D&D-based detectors, runtime was assessed in a technically reproducible Docker container with assigned memory of 32 GB and computational resources of 10 cores of an i7-1265U CPU at 2700 MHz that runs MLflow. While these methods were implemented in Python, established thresholding-based detectors in fNIRS were implemented in Matlab but calculated on the same machine that runs the docker container.

Generally, lower training and inference times were observed on the N21 dataset compared to the R22 dataset (see Table S6 and Table S7). This difference can be attributed to the smaller size of the N21 dataset (N=640) compared to the R22 dataset (N=5984). Further, the dataset size for training is larger than for inference (60% vs. 40%). In summary, NiReject provides a run-time-efficient implementation, especially in the category of semi-supervised detectors.

Table S6 shows the runtime of the established thresholding-based detectors which are based on existing Matlab implementations. Since some detectors are implemented together in a joint function, as seen in the Phoebe toolbox, their runtime is provided together. Particularly SNR and CoV showed lower runtime compared to more complex methods such as Phoebe.

Table S6 Runtime results: Showing means and standard deviations of runtimes across established detectors in Matlab.

Type	Regime	Models	Train Time (s) N21	Train Time (s) R22	Inference Time (s) N21	Inference Time (s) R22
Established	thresholding	CoV & SNR	21.20 ±0.30	25.67 ±0.17	14.16 ± 0.30	17.13 ±0.17
fNIRS detectors	thresholding	SCI & Phoebe & PeakPower	3655.31 ±39.94	4509.18 ±6.26	2456.97 ±39.94	3002.57 ±6.26
	thresholding	SignalLevel	43.06 ±0.71	116.10 ±0.32	28.67 ±0.71	77.28 ±0.32

Table S7 shows that HBOS (on first place) and PCA (on second place) exhibited the best training and inference times. Unsupervised NiReject also ranked among the most run-time-efficient algorithms. The efficiency of NiReject can be mainly attributed to the copula-based approach and its low computational overhead. Further,

incorporating priors reduces the complexity of the calculations, as the operations for tail selection and correction are reduced. Similar to KNN, the inference time for unsupervised NiReject is higher than the training time. For NiReject this is due to the application of the consistency loss and the processing of quality signatures to provide an interpretable user output.

Among semi-supervised methods, semi-supervised NiReject clearly outperforms all other detectors due to its efficient implementation and lower complexity compared to the autoencoder FEAWAD and the ensemble method XGBOD (Table S7). Unlike unsupervised NiReject, semi-supervised NiReject does not rely on consistency loss, resulting in reduced inference time (see main text Fig. 2).

Table S7 Runtime results: Showing means and standard deviations of runtimes across D&D, un-, and semi-supervised detectors in Python.

Type	Regime	Model	Train Time (s) N21	Train Time (s) R22	Inference Time (s) N21	Inference Time (s) R22
D & D	unsupervised	ABOD	9.66 ±19.54	44.38 ±18.89	2.21 ±0.16	25.59 ±0.79
	unsupervised	HBOS	0.18 ±0.01	0.32 ±0.06	0.01 ±0.00	0.06 ±0.01
	unsupervised	KNN	0.27 ±0.01	9.89 ±1.11	0.82 ±0.02	14.16 ±0.87
	unsupervised	LOF	0.39 ±0.02	12.02 ±0.96	0.21 ±0.01	7.81 ±0.65
	unsupervised	MCD	3.70 ±0.36	81.57 ±4.98	0.02 ±0.00	0.08 ±0.02
ML	unsupervised	CBLOF	13.02 ±25.48	66.48 ±24.20	0.07 ±0.03	0.22 ±0.05
	unsupervised	IForest	15.16 ±1.21	27.75 ±1.97	2.81 ±0.30	7.01 ±0.36
	unsupervised	OCSVM	0.78 ±0.04	49.74 ±4.25	0.21 ±0.02	14.65 ±1.14
	unsupervised	PCA	0.11 ±0.01	0.54 ±0.28	0.01 ±0.00	0.10 ±0.03
proposed	unsupervised	NiReject	0.27 ±0.01	1.15 ±0.06	1.08 ±0.04	3.57 ±0.19
ML	semi-supervised	FEAWAD	4390.71 ±841.51	7250.72 ±4432.63	78.01 ±35.90	149.66 ±118.09
	semi-supervised	XGBOD	109.32 ±39.42	946.85 ±121.55	21.01 ±1.30	392.49 ±63.32
proposed	semi-supervised	NiReject	14.39 ±1.18	44.40 ±2.71	0.66 ±0.06	3.00 ±0.23

Scalability of NiReject. To assess the scalability of NiReject, we conducted an experiment randomly sampling 100, 500, 1,000, 10,000, and 100,000 observations from R22 for assessing training time and the same number for assessing inference time. These datasets were strictly used for evaluating computation time without running MLflow tracking server used in experiments Q1-4. Figure S4 shows that NiReject scales nearly linearly with increasing sample size. However, for inference, semi-supervised NiReject shows a lower runtime for larger datasets. This is because the data augmentation procedure used for the consistency loss in unsupervised NiReject results in higher computational complexity.

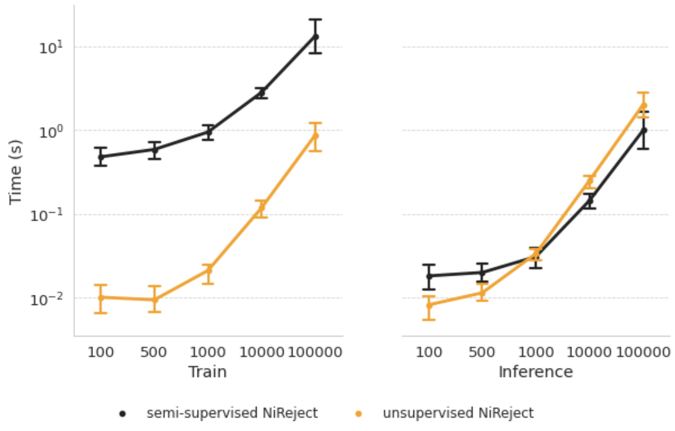

Fig. S4 Scalability of un- and semi-supervised NiReject. The line plots show the mean and standard error for train time (left) and inference time (right)**.**

4.3 Additional performance metrics

Since ROC-AUC values and Precision@n require continuous detection scores, we reported the performance of un- and semi-supervised detectors with continuous detection scores in Fig S3. These results are consistent with the interpretation in Sec 3.2 of the main text. Notably, Fig S5 suggests an increased ROC-AUC score of NiReject and

XGBOD compared to SCI. However, SCI also showed a high number of false positives (see Fig. S6). All detailed results (/source files) are available in the artifact storage.

(a)

Type	Regime	Model	ROC-AUC Scores N21 ($c = 9.38\%$)	ROC-AUC Scores R22 ($c = 4.22\%$)	Precision@n N21 ($c = 9.38\%$)	Precision@n R22 ($c = 4.22\%$)	AUC-PR N21 ($c = 9.38\%$)	AUC-PR R22 ($c = 4.22\%$)
D & D	unsupervised	ABOD	65.09 ±4.19	64.02 ±2.91	18.75 ±7.67	14.26 ±1.94	18.17 ±4.65	11.19 ±1.96
	unsupervised	HBOS	76.84 ±5.27	86.43 ±0.82	36.25 ±8.57	31.58 ±2.11	35.26 ±9.29	30.32 ±1.66
	unsupervised	KNN	69.86 ±3.13	66.04 ±2.47	19.58 ±6.82	15.35 ±2.25	21.30 ±6.02	12.31 ±2.17
	unsupervised	LOF	67.86 ±3.91	61.82 ±2.41	14.58 ±6.29	16.04 ±2.75	19.78 ±5.14	12.21 ±2.33
	unsupervised	MCD	74.11 ±4.25	73.70 ±1.43	22.92 ±6.87	11.58 ±1.62	29.03 ±7.20	11.57 ±1.35
ML	unsupervised	CBLOF	67.74 ±7.13	70.29 ±5.57	25.83 ±15.19	17.33 ±4.94	26.65 ±12.85	14.61 ±3.28
	unsupervised	IForest	72.38 ±4.33	87.16 ±0.96	32.08 ±8.11	20.89 ±3.69	30.36 ±8.51	22.22 ±2.46
	unsupervised	OCSVM	70.98 ±3.82	83.98 ±0.98	27.92 ±9.22	13.66 ±1.80	24.90 ±8.00	18.08 ±1.99
	unsupervised	PCA	71.49 ±4.09	87.68 ±0.88	33.75 ±8.21	15.05 ±1.53	31.26 ±10.04	22.44 ±2.24
proposed	unsupervised	NiReject	81.35 ±3.70	94.55 ±0.38	43.33 ±7.66	48.51 ±1.32	41.89 ±7.84	45.77 ±2.77
ML	semi-supervised	FEAWAD	75.08 ±5.75	95.15 ±1.18	44.17 ±6.57	59.50 ±4.82	46.11 ±7.36	57.39 ±6.84
	semi-supervised	XGBOD	79.94 ±3.66	97.75 ±0.49	44.17 ±6.57	69.31 ±2.29	50.04 ±4.69	75.66 ±2.46
proposed	semi-supervised	NiReject	83.96 ±3.41	97.34 ±0.31	50.00 ±4.81	67.13 ±2.87	54.01 ±4.94	74.44 ±1.67

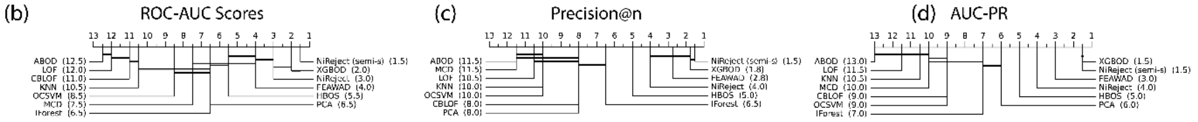

Fig. S5 Performance for continuous detection scores confirmed the main results: (a) Detection performance across detectors and datasets. (b-d) Bayesian difference diagram showing the average detector ranking (lower ranking indicates better performance). Thick horizontal lines connect groups of detectors with no evidence of practically relevant performance differences (CI = 90%, ROPE = [-1.0, 1.0]).

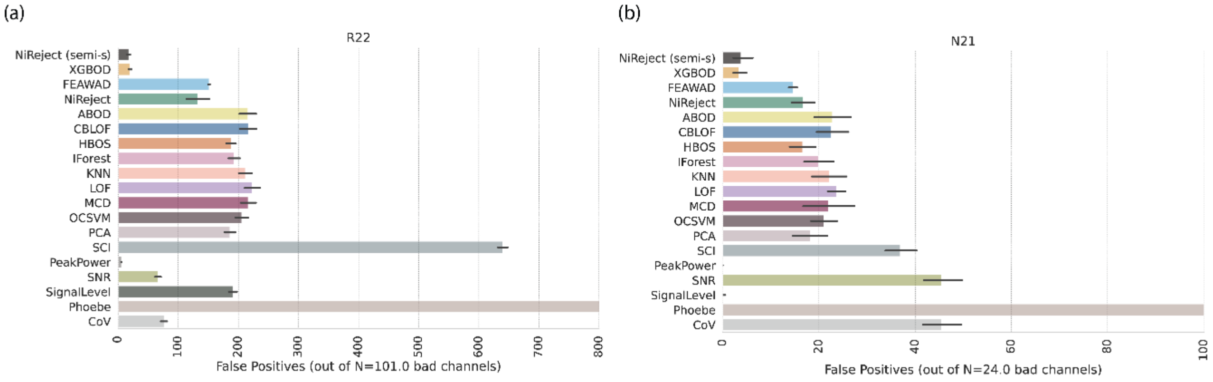

Fig. S6 False positives show limitations of thresholding-based approaches: (a) Number of false positives per detector on R22. The X-axis was limited to (0, 800) for better visibility. (b) Number of false positives per detector on N21. The X-axis was limited to (0, 100) for better visibility. Error bars represent the standard errors. It should be noted that Phoebe has a higher number of false positives as the axis limits.

Further, the Spearman correlation of precision across detectors showed low correlations among the semi-supervised detectors NiReject and XGBOD on the two real-world datasets (see Fig. S7). Since XGBOD resembles multiple unsupervised detectors (PCA, HBOS, KNN, IFOREST), one might expect XGBOD to show an increased correlation to these detectors. This was not the case because XGBOD was trained using rating information. Specifically, the low correlation of XGBOD indicates its sensitivity to rating information. This is in line with the observed performance gain compared to PCA, HBOS, KNN, and IFOREST. In contrast, FEAWAD showed higher correlations with unsupervised detectors, suggesting potentially lower sensitivity to rating information. XGBOD and NiReject showed a moderate correlation. We did not include peak power in this analysis since its precision was zero on N21.

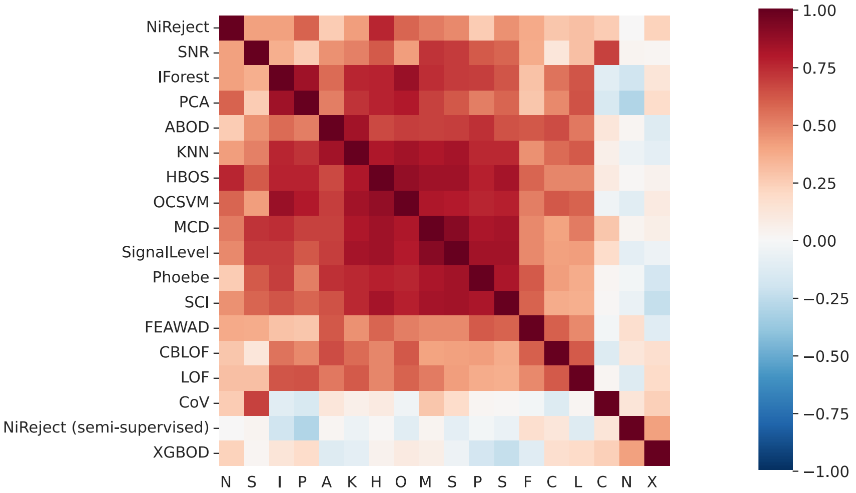

Fig. S7 Spearman correlation of precision across detectors. Both axes have the same order and color represents the correlation between two detectors.

5. Detector behavior under variations of bad channel phenomena: additional results

Figure S6 shows the performance profiles of all detection methods across 13 simulated dataset. All detailed results (/source files) are available in the artifact storage.

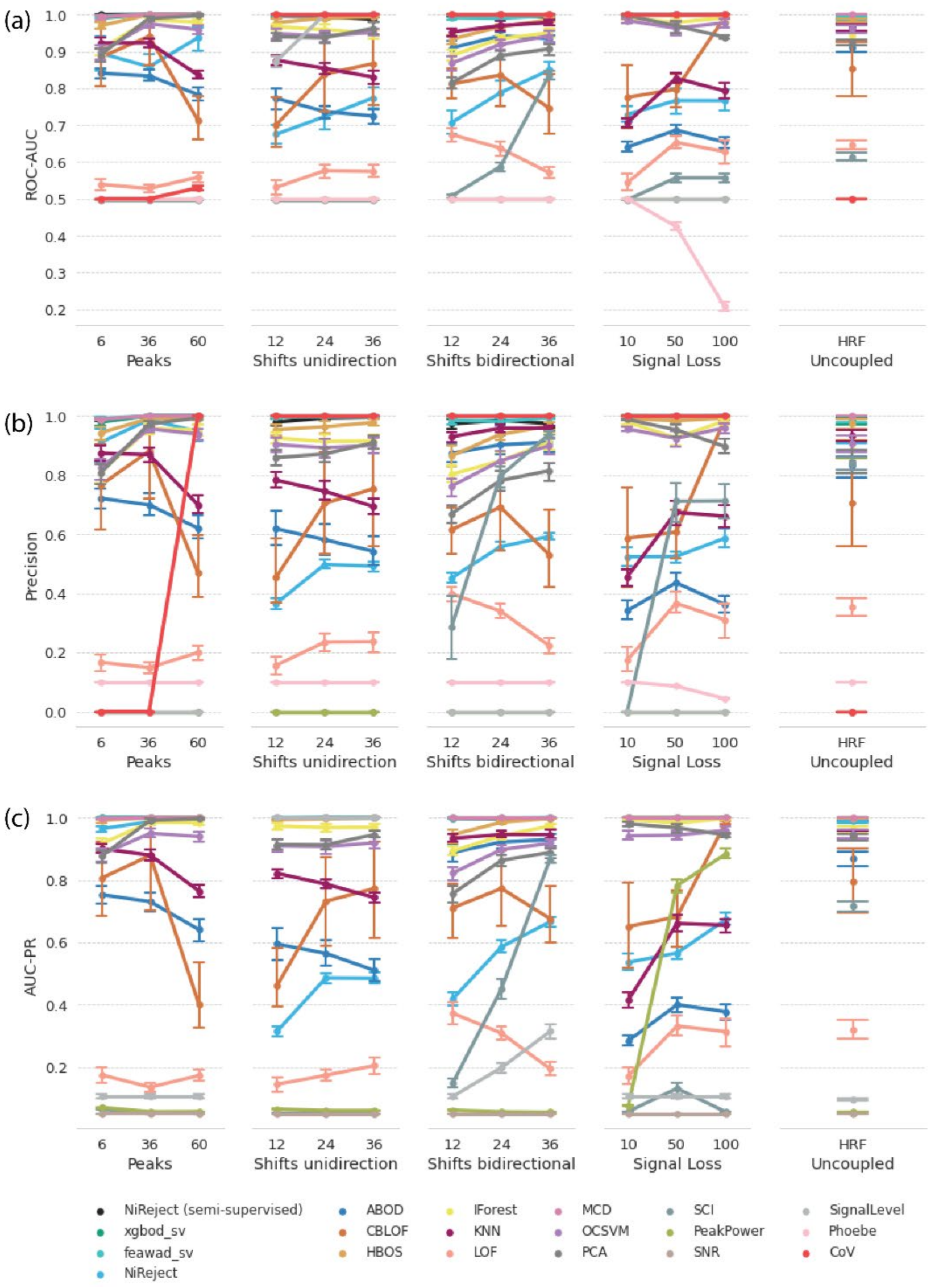

Fig. S8 Performance profiles across phenomena. The figure shows the (a) ROC-AUC, (b) Precision, and (c) AUC-PR of color-coded detectors on simulated datasets. Parameterizations of bad channel phenomena are depicted on the X-axis and performance is shown on the Y-axis with error bars representing the standard errors.

6. Detector behavior under variations of dataset characteristics: additional results

6.1 Varying contamination rates

The detector's behavior across varying contamination rates was assessed across multiple evaluation metrics (see Fig. S9). The ROC-AUC metric, which quantifies the trade-off between true positive rate and false positive rate, showed divergent trends among the algorithms. Specifically, SCI showed robust performance across most contamination rates, maintaining high ROC-AUC values. XGBOD displayed variable performance. While the ROC-AUC of NiReject (semi-supervised) increased with increasing contamination rates, the reverse trend was observed for most of the other detectors.

In terms of precision, NiReject (semi-supervised) emerged consistently as performant. Importantly, NiRejects stood out by a high increasing number of true positives and a low increasing number of false negatives, suggesting a good performance across contamination rates. The SCI also stood out by a high increasing number of true positives but, at the same time, a high increasing number of false negatives. Since the SCI labels a lot of signals as bad channels including most true bad channels, upsampling the bad channels will proportionally increase the number of true positives but also the number of false positives. All detailed results (/source files) are available in the artifact storage.

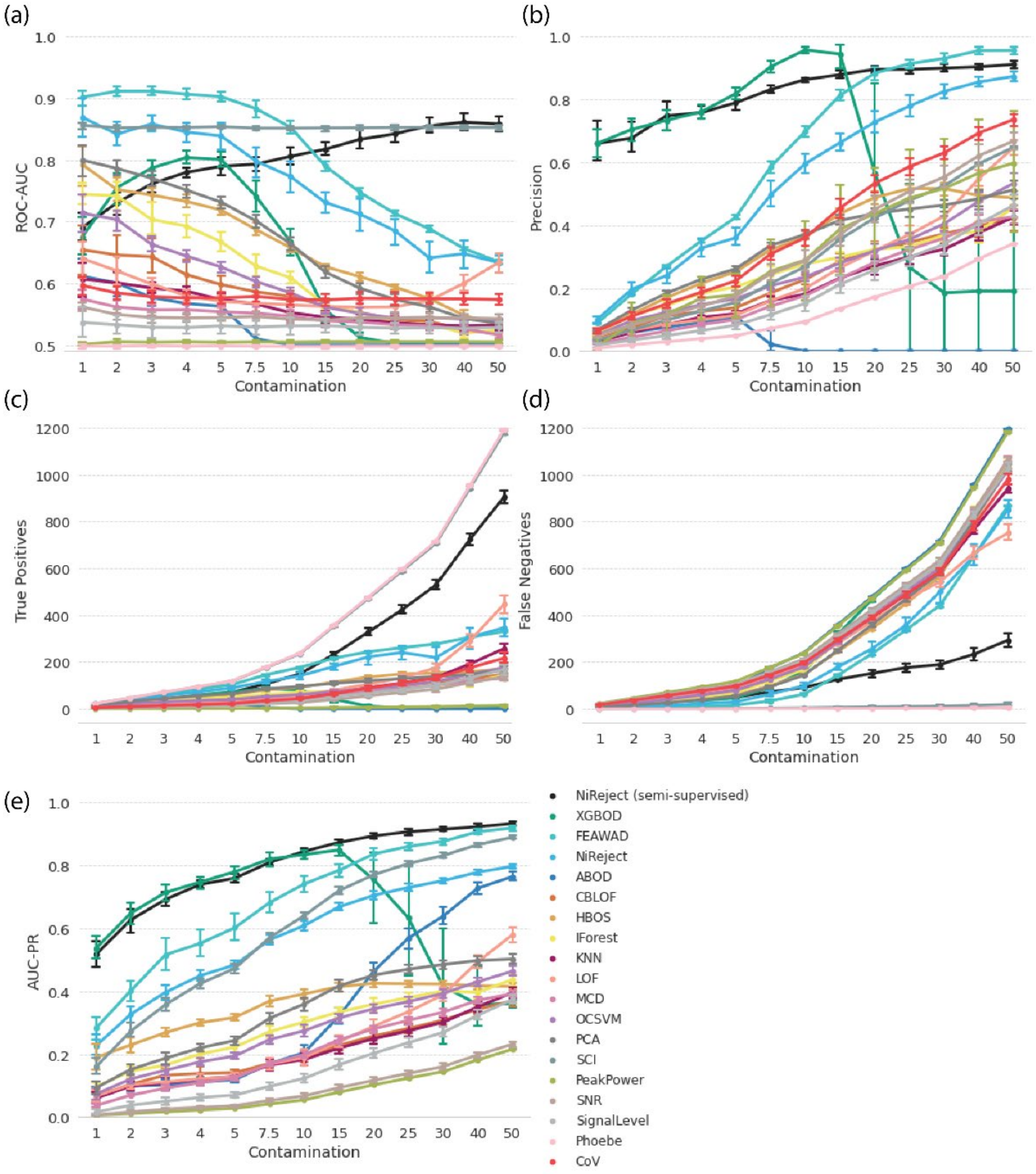

Fig. S9 Performance profiles across varying contamination rates. The figure shows the (a) ROC-AUC, (b) precision, (c) number of true positives, (d) number of false negatives, and (e) AUC-PR of color-coded detectors on simulated datasets with varying contamination rates. The performance metrics are depicted on the Y-axis, with error bars representing the standard errors. The X-axis represents the contamination rate.

6.2 Rating effort and annotation errors

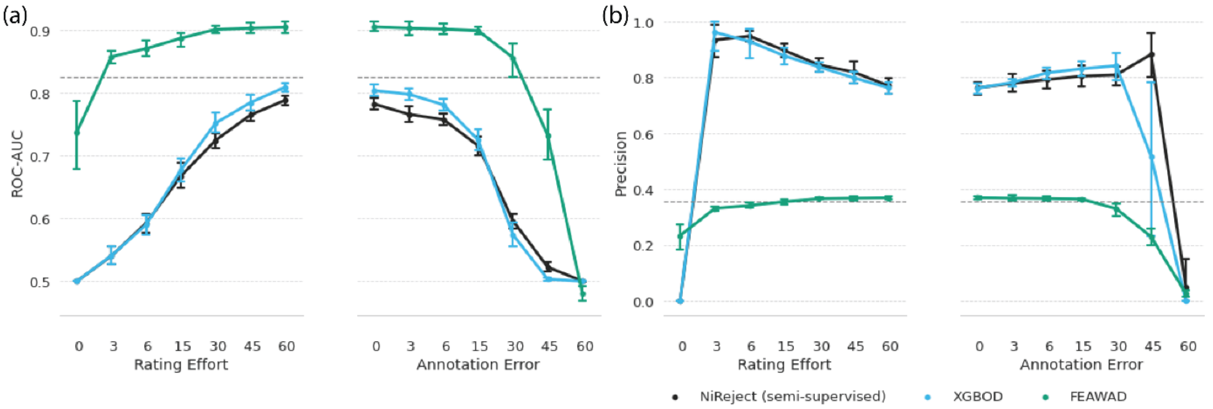

Fig. S10 Varying rating effort and annotation errors. (a) Shows ROC-AUC and (b) Precision to determine the critical number of rated bad channels for semi-supervised detectors. FEAWAD reached its performance maxima with $\gamma >= 6\%$, both NiReject and XGBOD approximately reached an asymptotic behavior with $\gamma >= 30\%$ rated bad channels. All semi-supervised detectors showed a strong performance drop if more than 15% of rated bad channels were rated incorrectly. Also, for semi-supervised NiReject, a decrease in ROC-AUC was observed after reaching 15% annotation error and a slight increase in precision. This implies an increased number of false negatives. When the annotation errors exceeded 45%, the precision of semi-supervised NiReject decreased drastically.

7. Details on empirical assessment of expert ratings

In the main text Sec. 3.5 we assessed the performance of a hybrid NiReject system on a simulated dataset of 100 subjects with a total of 2,500 signals. The first rater reviewed 533 (21.32%) signals, while the second rater reviewed 370 (14.8%) signals. The difference in the rated amount results from the requirement to rate 90 channels as bad to reach $\gamma = 30\%$. Signals with high, medium, and lower detection scores from unsupervised NiReject were presented to the raters in batches of 12 in randomized order. By comparing the raters' decisions against the simulated acceptable or bad channels (ground truth), the performance of both raters was assessed, as shown in Fig. S11.

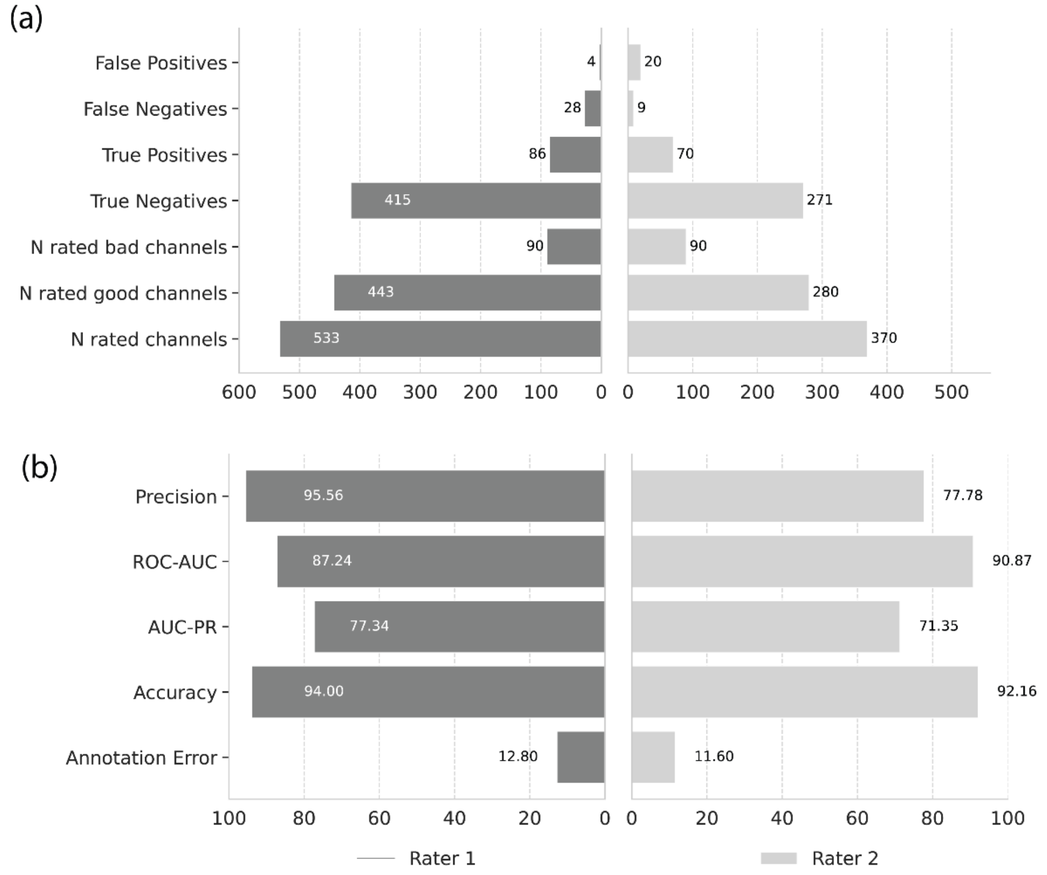

Fig. S11 Performance evaluation of two expert raters based on simulated fNIRS signals of 100 subjects. The pair plots show performance metrics for rater 1 (left) and rater 2 (right). (a) include counts of false positives, false negatives, true positives, true negatives, and total number of channels rated as acceptable or bad. (b) depicts ROC-AUC, AUC-PR, accuracy, annotation error, and annotation error specifically on known bad channels.
